# Proteomimetic Strategy for the Modulation of Intrinsically Disordered Protein MYC

**DOI:** 10.1021/jacs.4c18144

**Published:** 2025-04-08

**Authors:** Thu Nguyen, Seong Ho Hong, Paramjit Arora

**Affiliations:** Department of Chemistry, New York University, 100 Washington Square East, New York, New York 10003, United States

## Abstract

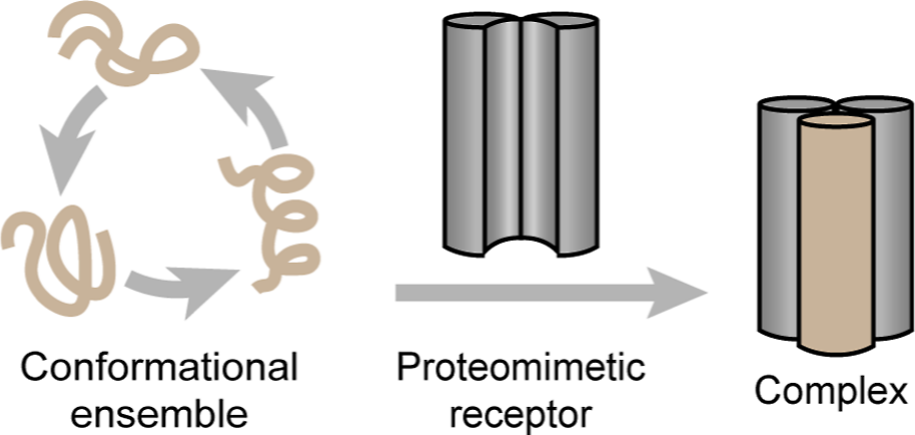

The difficulty in developing specific ligands for protein receptors is directly correlated to the presence of unique binding sites on the protein surface. Conformationally dynamic proteins increase the level of difficulty in ligand design, and the challenge is further exacerbated for proteins that are intrinsically disordered. Intrinsically disordered proteins (or IDPs) do not adopt a fixed three-dimensional shape until they bind their target; an absence of organized binding sites underscores the difficulty in developing synthetic ligands for these proteins. We hypothesized that one avenue for the development of binders for a disordered region would be to trap one of its thermodynamically accessible conformations in a receptor. Here, we show the application of this approach to MYC, which represents a critical therapeutic target but has not yielded small-molecule inhibitors due to its conformationally dynamic nature. MYC adopts a helical configuration when it binds to its cellular partner MAX. We rationally designed a proteomimetic scaffold to trap this conformation. We show that MYC can be directly engaged in both biochemical and cellular assays. Overall, this work demonstrates a general method to capture and trap intrinsically disordered proteins with a propensity to adopt α-helical conformations.

## Introduction

**Figure 1:**
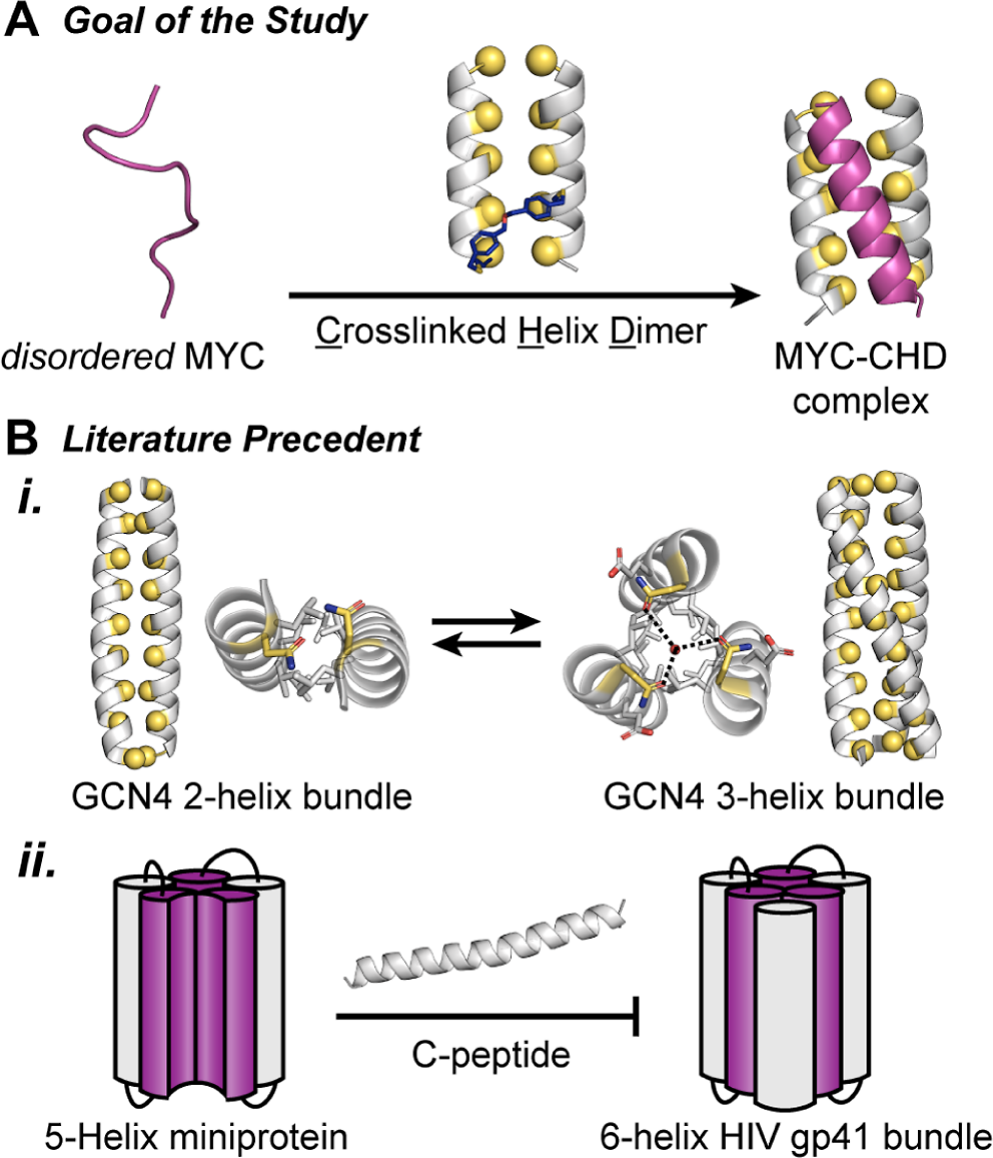
(A) Proposed strategy to trap disordered MYC^406–436^ sequence with a rationally designed cross-linked helix dimer receptor (CHD). (B) (i) GCN4 leucine zipper can populate either a dimeric or trimeric fold—with the dimer serving as a host for the third strand (PDB codes: 4DMD and 4DME). (ii) A 5-helix bundle can accommodate C-peptide from HIV gp41 to form a 6-helix bundle.

Traditional approaches to target proteins have relied on the paradigm that the unique 3D folds of proteins provide ligandable binding sites. A range of small-molecule drugs has been engineered or found to engage these binding pockets. However, estimates suggest that more than 85% of the human proteome lacks “druggable” pockets typically targeted by conventional small molecules.^1^ Intrinsically disordered proteins or regions (IDPs or IDRs) represent a significant challenge in drug discovery because they lack discrete binding sites.^2^ Additionally, IDPs or IDRs can undergo folding upon binding to their binding partners.^3^ Several strategies to develop ligands for IDPs have been considered that include NMR and computational approaches to identify targetable conformational ensembles to screening of high-throughput compound libraries in functional assays.^4−6^ Here, we tested a rational design approach to target IDRs: we hypothesized that the native secondary structure conformation adopted by bound IDRs may be trapped as part of a tertiary structure conformation (Figure 1A).

We applied our design strategy to MYC, an intrinsically disordered transcription factor implicated in oncogenesis.^7^ Various transcription factors are classified as intrinsically disordered until they dimerize or are recognized by DNA or a coactivator. Transcription factors belonging to the basic helix loop helix (bHLH) and leucine zipper (bZIP) family of proteins characteristically display this disorder-to-order transition upon binding to their partners. The oncogenic MYC protein, a well-characterized member of the bHLH/bZIP family, has emerged as a challenging target, due to its disordered conformation.^8,9^ MYC exists in an ensemble of conformational states and engages in transient interactions with multiple binding partners. Identification of selective ligands for MYC has been difficult because MYC lacks ligandable sites for traditional small molecules.^10−12^ Previous peptide- and protein-based attempts to target or mimic MYC have yielded OMOMYC, a 91-amino acid mini-protein to sequester MYC and synthetic transcriptional repressors that occupy E-box promoters.^13,14^

We focused on trapping the helical configuration adopted by MYC in its bound state with a synthetic receptor. Prior efforts with coiled coils as hosts for helical peptides provide a framework for our studies.^15,16^ The analysis of the folding behavior of the leucine zipper domain of GCN4 has shown that this coiled coil can switch between dimeric and trimeric states, suggesting that a 2-helix bundle can host a third helix (Figure 1B).^17−19^ In a classical study, Kim et al. demonstrated that a designed 5-helix gp41 protein bundle could effectively capture an HIV C-peptide helix with high affinity to form a 6-helix bundle (Figure 1B).^20^ DeGrado and co-workers have extensively explored synthetic receptors consisting of coiled coil topologies, including the computational design of binding sites for small molecules.^21,22^ The challenge in the design of synthetic receptors for intracellular proteins is that the receptor must be cell-permeable. We have recently described a strategy to cross-link short peptides to access a helix dimer.^23−25^ Our strategy utilizes a covalent bond in place of an ionic interaction, typically found between coiled dimers, and requires sculpting of the helix interface with an optimal knob-into-hole (KIH) packing.^23,24^ These principles led us to the design of cross-linked helix dimers (CHDs) as minimal proteomimetics with the potential to modulate intracellular protein–protein interactions. In previous efforts, we developed CHDs as structural mimics of proteins Sos and NEMO to inhibit cellular signaling mediated by these proteins.^26,27^ Here, we investigated whether the CHDs could serve as cell-permeable synthetic receptors for disordered proteins that have a propensity to adopt helical conformations.

## Results

### Design of MYC-Specific Receptors

To develop MYC-specific receptors, we focused on the potential of this protein to form coiled coil assemblies. A trove of literature analyzing the stability and formation of coiled coils suggests that in the absence of specific features, trimeric coiled coils are more stable than dimeric coiled coils;^28^ for example, the trimer is the default oligomerization state for peptide sequences that feature hydrophobic residues at the “a” and “d” positions of the heptad repeat, although polar interactions may populate dimers or tetramers.^18,28^ Indeed, dimeric coiled coils have previously been envisioned as receptors for helical peptides to obtain trimers.^22^ Lastly, the fluctuation of the GCN4 sequence between the dimer and trimer states may be envisioned as the result of a dimeric coiled coil hosting a third strand (Figure 1B). We conjectured that an appropriately modified dimeric MAX scaffold may trap MYC to form a stable heterotrimeric complex.

**Figure 2:**
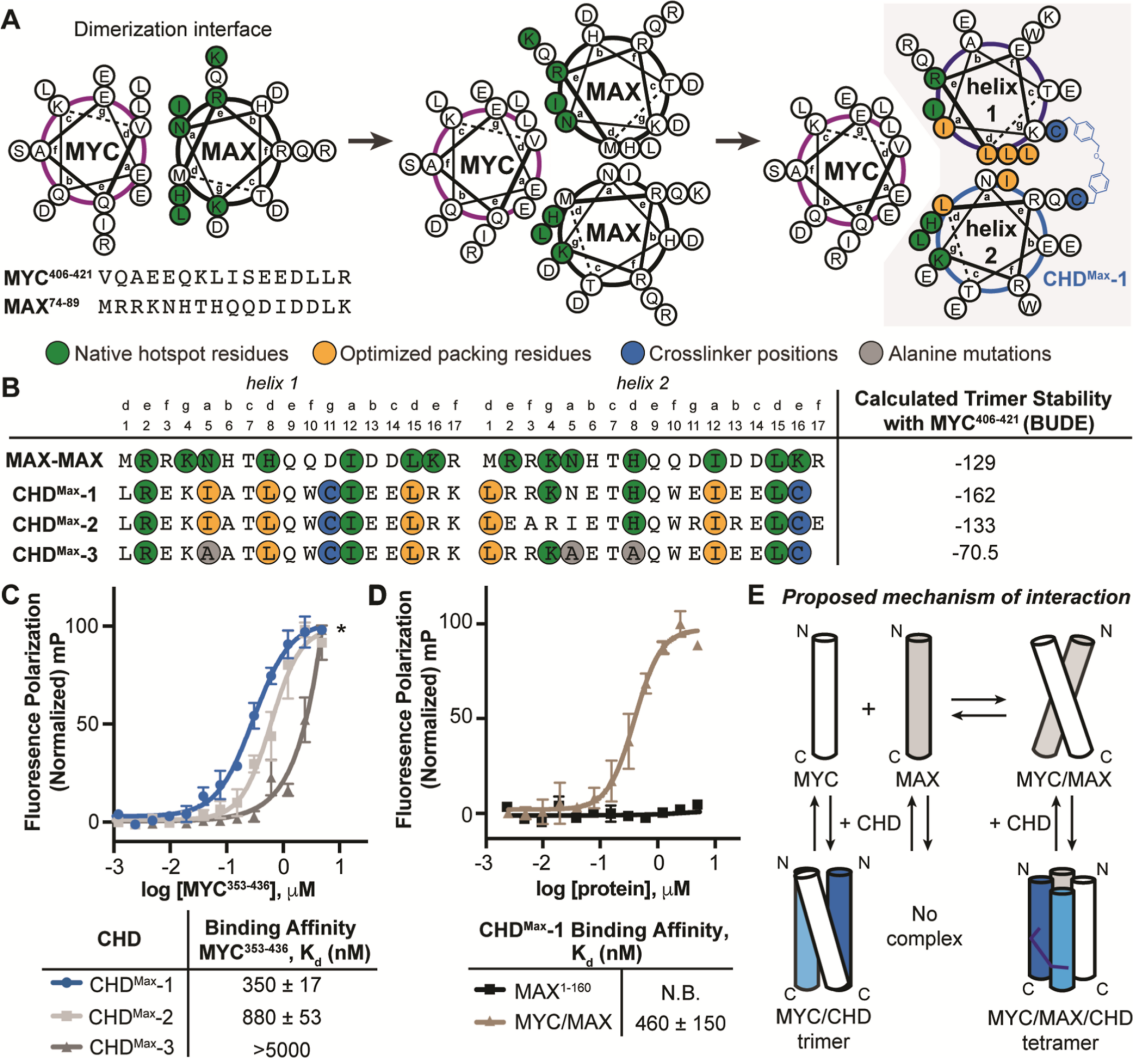
Rational design, biophysical characterization, and stability of MAX-derived cross-linked coiled coils. (A) Helical wheel diagrams depicting the native MYC/MAX dimerization interface (left), a proposed trimer where dimeric MAX may host a MYC sequence (middle), and designed trimeric interaction of MYC with MAX-derived constrained helix dimer (right). (B) Peptide sequences of designed compounds. We utilized CCBuilder, a computational tool that predicts coiled coil stabilities using the BUDE (Bristol University Docking Engine) force field. The BUDE energies of CHDs in complex with MYC^406–421^ are listed. (C) Binding affinities of fluorescein-labeled CHDs for His_6_-MYC^353–437^ were analyzed in fluorescence polarization (FP) assays. *indicates maximum concentration of His_6_-MYC^353–437^ possible in the assay due to the observed aggregation at higher values. (D) FP assay to assess binding between the fluorescein-labeled CHD^Max^-1 and His_6_-MAX, or the MYC/MAX complex. (E) Schematic representation of the proposed model for the interactions of CHD^Max^-1 with MYC alone and the MYC/MAX complex.

We began the design of a MYC binder by analyzing the crystal structure (PDB: 1NKP) of the native MYC/MAX leucine zipper with computational alanine mutagenesis scanning to identify residues from MAX that make important contribution to the heterodimer stability. This analysis showed that seven MAX residues (Arg75, Lys77, Asn78, His81, Ile85, Leu88, and Lys89) are important for MYC binding (Figure 2A, Supporting Information Table S1).^29,30^ The designed cross-linked MAX dimer (**CHD**^**Max**^**-1**) thus encompasses MAX^74–89^ residues to trap MYC (Figure 2A) based on the high-resolution structure of the MAX homodimer (PDB:1AN2).^31^ To cross-link **CHD**^**Max**^, we incorporated a dibenzyl ether linker in place of a salt-bridge between Asp11 on helix 1 and Lys16 on helix 2; these residues were substituted with cysteine which was then alkylated to incorporate the thio-ether-based cross-linker (Supporting Information Figure S9).^25^

The stability of coiled coils is maintained by knob-into-hole (KIH) packing at helix interfaces, while critical polar and ionic contacts provide specificity for coiled coil formation. In constructing minimal coiled coil mimics, we have shown that optimal KIH packing residues are critical for restricting conformational heterogeneity.^24,32,33^ Analysis of the native MAX homodimer shows that the stability of the dimer complex is dependent on interhelical hydrogen bonding. We optimized the KIH packing at the helical interface to access a stable minimally dimeric motif. We introduced Ile and Leu at “a” and “d” positions of helix 1 to develop a parallel homodimer. **CHD**^**Max**^**-1** retains important ionic residues from MAX for interactions with MYC, including His6 of MAX on helix 2 to potentially engage two glutamic acids at the “a” position of MYC. Additionally, Met1 at the “d” position was swapped with leucine to avoid thioether oxidation during the synthesis. Aspartic acids on noninteracting “c”, “f”, and “g” positions were also mutated to glutamic acids to avoid undesired aspartimide formation. His6 and Gln10 at positions “b” and “f” were mutated to increase intra- and interstrand salt bridge interactions and the conformational stability of the helix dimer.^34^ Lastly, we swapped Lys16 and Arg17 in native MAX with Arg16 and Lys17, respectively, in helix 1 with the expectation that Arg16 can potentially form ionic interactions with MYC glutamic acid residues 409 and 416 at “g” positions.

We computationally assessed the rationally designed CHDs with CCBuilder 2.0, a parametric α-helical coiled coil prediction tool developed by Woolfson and Wood, to gauge the free energy of binding between helices (Table S2).^35^ CCBuilder utilizes BUDE (Bristol University Docking Engine) force field to predict the stability of coiled coils. This computational analysis suggests that the MYC^406–421^–**CHD**^**Max**^**-1** complex is potentially substantially more stable than the putative binding of MYC^406–421^ with the native MAX^74–89^ dimer, as indicated by the BUDE values of −162 for **CHD**^**Max**^**-1**/MYC versus −129 BUDE predicted for (MAX)_2_/MYC complexes (Figure 2B). We experimentally measured the binding affinity of fluorescein-labeled **CHD**^**Max**^**-1** (**FITC–CHD**^**Max**^**-1**) for recombinant human MYC^353–437^ protein using the FP assay. **FITC–CHD**^**Max**^**-1** exhibited a nanomolar binding affinity for MYC, *K*_d_ = 350 ± 17 nM (Figure 2C). We next interrogated if the exchange of helix 2 Asn5 residue in **CHD**^**Max**^**-1** with an isoleucine group provides further stability to the construct, potentially due to better KIH packing. CCBuilder predicts that this mutation would lead to a less stable complex when the analogue engages with MYC (Figure 2B). Fluorescein-labeled **CHD**^**Max**^**-2** was synthesized and tested for binding to MYC. **CHD**^**Max**^**-2** bound MYC with roughly 3 times lower affinity (*K*_d_ = 880 ± 53 nM) than **CHD**^**Max**^**-1**, in agreement with the CCBuilder prediction. We also designed **CHD**^**Max**^**-3** as a negative control. **CHD**^**Max**^**-3** shares structural and sequence similarity with **CHD**^**Max**^**-1** but contains three alanine mutations in place of important MYC-binding residues: helix 1 residue Ile-5, and helix 2 residues Ile-5 and His-8 (Figure 2B). CCBuilder calculations predict that the complex of **CHD**^**Max**^**-3** with MYC is less stable compared to the constructs with **CHD**^**Max**^**-1** and -**2**. This prediction was confirmed in our FP assay; **FITC–CHD**^**Max**^**-3** bound MYC with weak affinity (*K*_d_ > 5000 nM) and constitutes a negative control for further studies. The MYC^353–437^ protein used for the binding assays shows aggregation above 5 μM concentration, precluding the accurate assessment of the binding constant for weaker binders.

### Assessment of **CHD**^**Max**^**-1** for MYC Complexation

We sought to determine whether our lead compound **CHD**^**Max**^**-1** is specific for MYC or can also complex with other peptides capable of forming coiled coil complexes. We analyzed the binding of **FITC–CHD**^**Max**^**-1** with MAX in the FP assay—no binding was observed under the assay conditions (Figure 2D). This result is supported by the predicted BUDE analysis of the **CHD**^**Max**^**-1**/MAX complex (Table S2). Although, **CHD**^**Max**^**-1** does not bind MAX, we were surprised to learn that it can engage the preformed MYC/MAX heterodimer with nearly equal affinity as MYC alone (Figure 2D). Together, the binding data suggest that **CHD**^**Max**^**-1** can engage MYC as a trimer and assemble with the MYC/MAX heterodimer to form a tetramer (Figure 2E). A tetramer helix wheel assembly model and a computational prediction from CCBuilder are included in the Supporting Information, Table S3 and Figure S2.

To further support the proposed assemblies with **CHD**^**Max**^**-1**, we used an established thiol–disulfide exchange assay to monitor the equilibrium between disulfide-bonded species (Figure S4). This assay has been used extensively to examine the preferential association and helix orientation specificity of coiled coil peptides.^18,36^ First, we installed an N-terminal Cys-Trp-Gly-Gly linker to the synthetic leucine zipper domains of MYC and MAX (MYC-LZ and MAX-LZ) for disulfide bond formation (Table S4). Following incubation for 48 h in redox buffers, the disulfide-bonded species were separated and quantified by reverse-phase high-performance liquid chromatography (RP-HPLC). In agreement with our FP analysis, in the presence of equimolar **CHD**^**Max**^**-1**, we observed nearly 2-fold increase in MYC–MAX heterodimerization, suggesting that **CHD**^**Max**^**-1** also has a binding affinity toward the MYC–MAX complex (Figure S4). Although **CHD**^**Max**^**-1** does not interact with MAX in the absence of MYC, it is possible that **CHD**^**Max**^**-1** associates with the MYC/MAX complex through two pathways: (1) **CHD**^**Max**^**-1** binds to MYC first, followed by assembly with MAX or (2) **CHD**^**Max**^**-1** associates with preformed MYC/MAX dimers.

**Figure 3:**
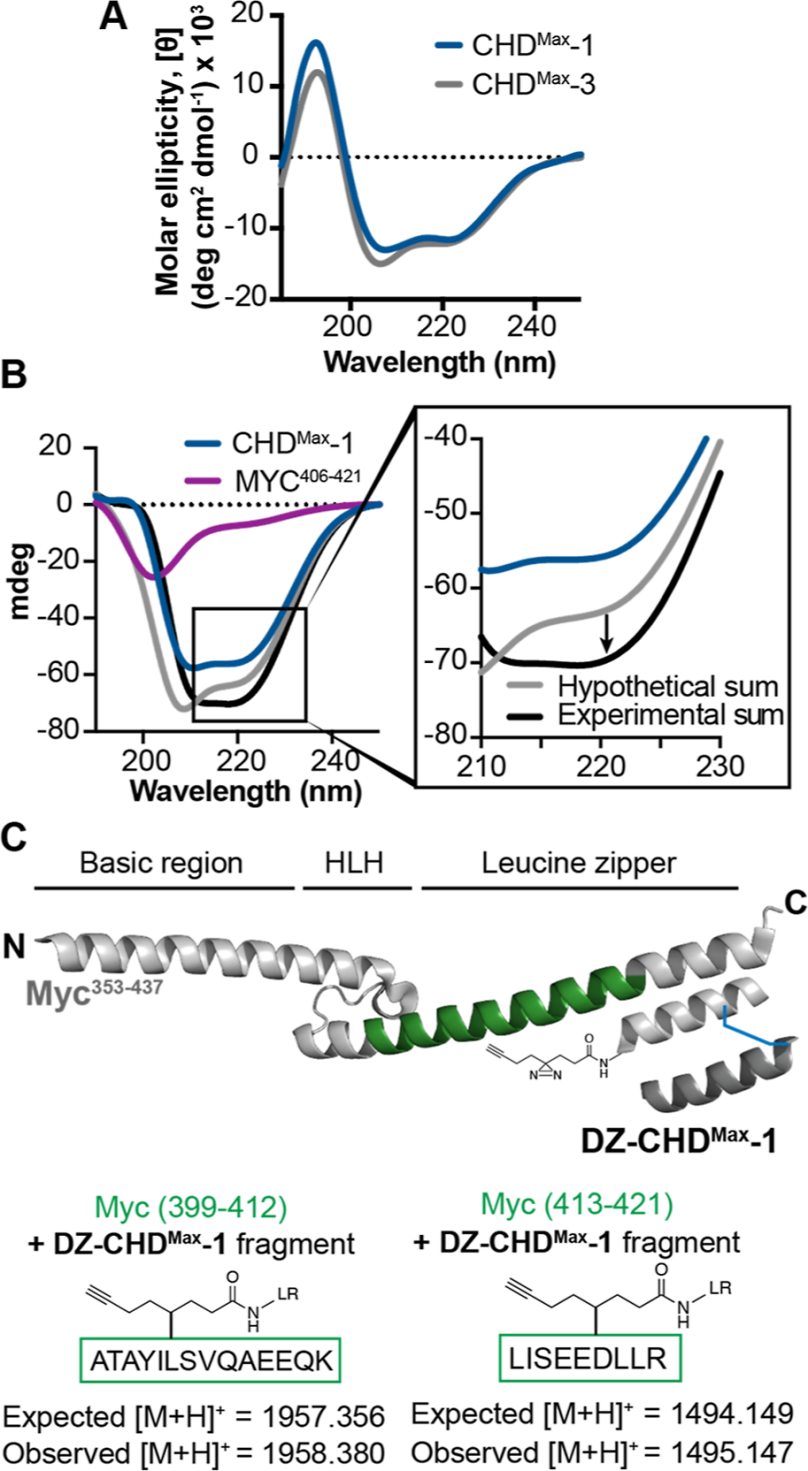
(A) The conformation of the CHDs was evaluated with CD in 0.1× PBS buffer at 20 μM peptide concentration. (B) Titration of 1:1 MYC^406–421^ and **CHD**^**Max**^**-1** indicates complex formation (100 μM peptide concentration). (C) The **CHD**^**Max**^**-1** binding site on MYC was analyzed with a photo-cross-linking reaction.

Our hypothesis is that CHDs can trap and stabilize disordered segments into helical configurations. We used circular dichroism (CD) spectroscopy to determine if MYC becomes helical upon binding **CHD**^**Max**^**-1**. The CD spectra of **CHD**^**Max**^**-1** and **-3** were obtained in 0.1× PBS buffer at pH 6.7. Both CHDs displayed spectra indicative of a helical conformation with a maximum at 190 nm and double minima at 208 and 222 nm (Figure 3A). In contrast, MYC^406–421^ shows a signal with a minimum at 190 nm, as expected of a random coil. Addition of one equivalent of **CHD**^**Max**^**-1** to MYC^406–421^ results in a strong change in intensity of 222 nm compared to the sum of two independent spectra of MYC^406–421^ or **CHD**^**Max**^**-1** (Figure 3B). The enhancement in the CD signal observed suggests that the conformational change of MYC to a more ordered structure occurs upon binding to **CHD**^**Max**^**-1**. To assess the increase in the helicity of MYC^406–421^, we conducted 2,2,2-trifluoroethanol (TFE) titration studies. Enhancement in peptide helicity as a function of increasing TFE concentration in water has been extensively studied, and it has been shown that peptide helicity reaches a maximum in 30–40% aqueous TFE solutions.^37,38^ The mean residue ellipticity of the peptide in this %TFE range has been thought to represent the maximum helicity for a peptide. TFE titration of MYC^406–421^ shows that maximum helicity is reached at 30% TFE, while the CD spectra of **CHD**^**Max**^**-1** showed minimal change upon the addition of 30% TFE (Figure S5). This analysis suggests that **CHD**^**Max**^**-1** is already fully helical in the aqueous buffer and that MYC^406–421^ reaches approximately 50% of its maximum helicity upon binding to **CHD**^**Max**^**-1**. We also performed titration of **CHD**^**Max**^**-1** with MAX^74–89^ under similar conditions and observed no change in the CD signal (Figure S6A), demonstrating no complex formation between MAX^74–89^ and **CHD**^**Max**^**-1**. The negative control of **CHD**^**Max**^**-3** results in no complex formation with MYC^406–421^, as expected (Figure S6B), consistent with a weaker BUDE energy for MYC/**CHD**^**Max**^**-3** as predicted.

The binding and CD assays show that the lead CHD can bind MYC with high affinity and induce the desired conformational change. In our FP binding assay, we used recombinant MYC protein (MYC^353–437^). We designed the CHD to localize onto a specific region of MYC spanning residues 400–430. To determine if the lead receptor binds to the desired MYC segment, we synthesized a **CHD**^**Max**^**-1** analogue with a diazirine handle, **DZ**-**CHD**^**Max**^**-1**, to photo-cross-link the MYC region engaged by the receptor. Treatment of **DZ**-**CHD**^**Max**^**-1** with MYC^353–437^ under UV irradiation, followed by trypsin cleavage, provided leucine zipper MYC fragments covalently linked by **DZ**-**CHD**^**Max**^**-1**, as identified by mass spectrometry analysis (Figure 3C, S7). This analysis shows that the designed CHD can engage MYC at the predicted site.

### Complexation of MYC by **CHD**^**Max**^**-1** In Cellulo

**Figure 4:**
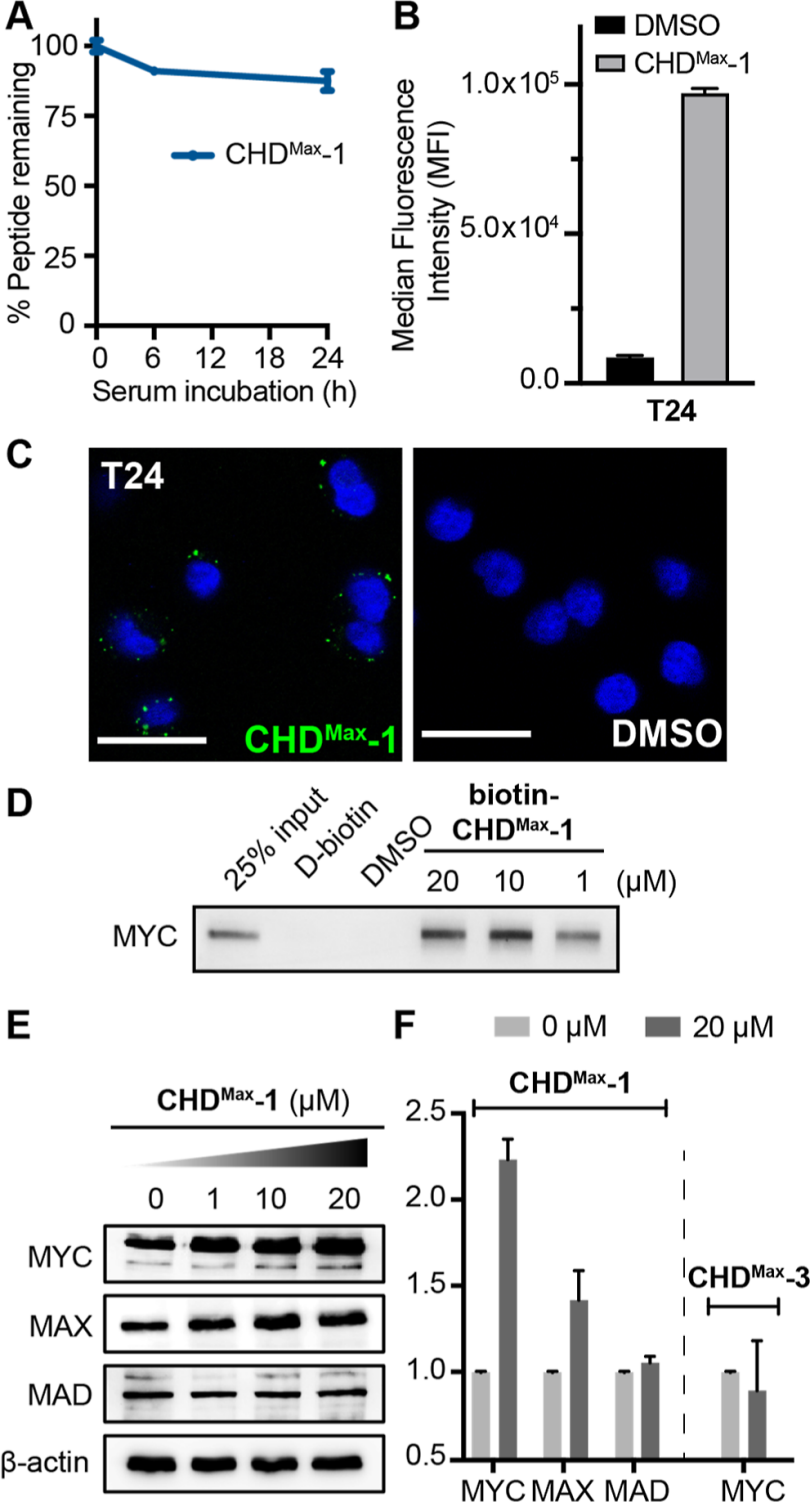
(A) Proteolytic stability of **CHD**^**Max**^**-1** in 25% FBS. Error bars are mean ± SD of biological replicates. (B) Flow cytometry quantification of fluorescently labeled **CHD**^**Max**^**-1** or DMSO in T24 cells. (C) Live-cell fluorescence imaging of Hoechst-stained T24 cells incubated with fluorescein-labeled **CHD**^**Max**^**-1** or DMSO for 4 h. Scale bar represents 10 μm. (D) Western blot analysis on endogenous MYC protein after **biotin-CHD**^**Max**^**-1** pull-down in T24 lysate. (E) Treatment of T24 cells with increasing concentrations of **CHD**^**Max**^**-1** for 24 h resulted in increasing protein levels of MYC and MAX but not MAD1. (F) Relative protein levels of MYC/MAX/MAD1 normalized to β-actin from Western blot in D.

Encouraged by the in vitro results, we evaluated the potential of **CHD**^**Max**^**-1** to form a complex with MYC in cellulo. We first assessed the ability of CHDs to resist proteolytic degradation in fetal bovine serum (FBS). We monitored the rate of peptide degradation under the treatment of 25% FBS over 24 h by RP-HPLC, with tryptophan as an internal standard. **CHD**^**Max**^**-1** exhibits exceptional resistance to proteolysis where roughly 80% of the initial peptide remained intact after 24 h (Figure 4A). This result is consistent with our previous studies showing that conformationally stabilized CHDs are highly resistant to enzymatic proteolysis.^26,27^ After validating the serum stability of **CHD**^**Max**^**-1**, we tested its potential to enter live cells. We chose T24 cells, a bladder cancer cell line, for these studies because these cells express a high level of MYC.^39^ We assessed the ability of fluorescein-labeled **FITC–CHD**^**Max**^**-1** to enter T24 cells using confocal microscopy and observed overlapped fluorescein and Hoechst nuclear stain signals, showing internalization of **FITC–CHD**^**Max**^**-1** (Figure 4C). We used flow cytometry to quantify the internalization of **FITC–CHD**^**Max**^**-1**, and this compound showed enhanced internalization as compared to DMSO control (Figure 4B).

To examine the engagement of endogenous MYC, we synthesized a biotinylated derivative of **CHD**^**Max**^**-1**, **biotin-CHD**^**Max**^**-1,** and evaluated its complexation with the MYC protein using pull-down experiments. In the nuclear extract of the T24 cell line, we found that **biotin-CHD**^**Max**^**-1** can engage endogenous MYC effectively at 1 μM concentration (Figure 4D). Binding and stabilization of MYC is expected to prevent its protein degradation in cells.^40^ To determine if the addition of **CHD**^**Max**^**-1** modulates endogenous MYC levels, we treated T24 cells with an increasing concentration of the compound. Following 24 h incubation, we observed a significant increase in MYC as well as MAX protein levels by Western blots in a dose-dependent manner (Figure 4E). The increased levels of both proteins are consistent with our model that **CHD**^**Max**^**-1** can bind to MYC alone and the MYC/MAX heterodimer. Importantly, the treatment of alanine control **CHD**^**Max**^**-3** had no effect on MYC or MAX protein levels (Figure 4F and S8A). We repeated the experiment with **CHD**^**Max**^**-1** treatment in another cell line (HCT-116 cells) and observed similar effects in the MYC and MAX stabilities (Figure S8B). Lastly, we tested the specificity of **CHD**^**Max**^**-1** for MYC as the monomer and MYC/MAX heterodimer by blotting against MAD1, a MYC antagonist that forms a heterodimer with MAX.^41^ We found that **CHD**^**Max**^**-1** treatment had no effect on the MAD1 protein level, indicating that the designed proteomimetic is specific for MYC or MYC/MAX (Figure 4F).

## Conclusions

The goal of this study was to test the hypothesis that proteomimetics can be rationally designed to trap disordered peptides. Building on the classical analysis of coiled coil peptides, we asked whether a helix dimer can be developed to complex a disordered intracellular domain and form a three-helix bundle. We developed cross–linked helix dimers (CHDs) to trap MYC—an oncogenic protein that has been difficult to target with small molecules. MYC may be bound with linear peptides that mimic its binding partner MAX; however, the advantage of CHDs over linear peptides is that the cross-linked constructs can be designed to be resistant to proteases and have been shown to be cell-permeable. We rationally designed **CHD**^**Max**^**-1** to serve as a cellular receptor for MYC. Our results show that this construct stabilizes MYC in cellulo by preventing its protein degradation. A similar strategy to trap IDRs was recently described by Baker et al.^42^ This group used computational design to develop binders for the Aβ42 peptide to inhibit fibril formation in Alzheimer’s disease. Together, these studies demonstrate that transient IDRs may be trapped by designed proteomimetic receptors. In summary, we present a general approach for designing a minimal proteomimetic scaffold to modulate IDRs that have an α-helix-forming propensity. In ongoing studies, we are testing the potential of the receptor to regulate MYC-mediated signaling. The overall goal of this program is to assess the biophysical and structural determinants for helical stabilization of a range of intrinsically disordered sequences. The results of these studies will be reported in due course.

## Supporting Information Available

Synthesis, characterization, and binding affinity analysis of CHDs (PDF)
