# Supplementary material for: Proteomimetic Strategy for the Modulation of Intrinsically Disordered Protein MYC
Source: J Am Chem Soc. 2025 Apr 8;147(16):13296–302. doi: 10.1021/jacs.4c18144 (PMC12022973; doi:10.1021/jacs.4c18144)
Supplement: Supplementary file 1 — ja4c18144_si_001.pdf [file ja4c18144_si_001.pdf]

Supporting Information

A Proteomimetic Strategy for Modulation of Intrinsically Disordered Protein MYC

Thu Nguyen‡, Seong Ho Hong‡, Paramjit Arora*
Department of Chemistry, New York University, 100 Washington Square East, New York, NY 10003

Table of Contents

	Page #
Supporting Figures and Tables	S2-S10
Supporting Methods and compound characterization	S11-S21
References	S22

Supporting Tables and Figures

Table S1. Computational alanine scanning for determination of hot-spot residues in the MYC-MAX complex (PDB 1NKP).

MYC LZ domain (406-425): VQAEEQKLISEEDLLRKRRE

MAX LZ domain (74-93): MRRKNHTHQQDIDDLKRQNA

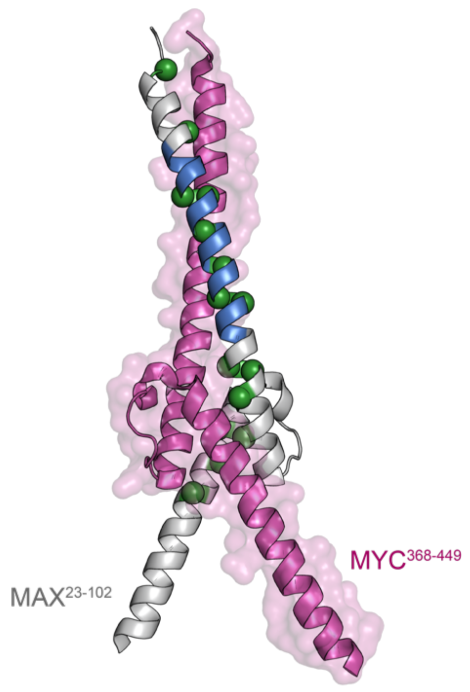

MAX residue	ΔΔG (kcal/mol) RosettaAlaScan	ΔΔG (kJ/mol) BUDEAlaScan
M 74	0.90	3.4
**R 75**	**1.54**	**2.9**
R 76	–	0
**K 77**	**0.09**	**5.2**
**N 78**	**0.62**	**6.0**
H 79	–	0
T 80	–	0
**H 81**	**1.84**	**6.1**
Q 82	0.02	1.2
Q 83	–	-
D 84	-0.07	0
**I 85**	**1.80**	**5.6**
D 86	–	0
D 87	–	0
**L 88**	**1.80**	**5.0**
**K 89**	**0.12**	**4.7**
R 90	–	-
Q 91	0.74	1.8
N 92	0.67	3.6
A 93	–	-

Figure S1. Alanine scanning mutagenesis of Myc$^{368\text{-}449}$ (magenta)-Max (gray) complex (PDB: 1NKP) reveals critical Myc binding residues (green spheres). Although, the energetically important residues for heterodimerization are dispersed over large Max surface, a cluster of these residues (blue helix) thereby provides a lead domain for rational design of Myc ligands. Computational analysis was performed with Rosetta[1] and BAlaS (BUDEAlaScan).[2]

Table S2. BUDE energy predicted by CCBuilder 2.0 program.

Dimer species	BUDE energy	Trimer species	BUDE energy
MYC/MYC	**25.4**	MYC/MAX/MAX	**-128.9**
MYC/MAX	**-62.9**	MYC/CHD^{Max}-1	**-161.9**
MAX/MAX	**-54.9**	MAX/ CHD^{Max}-1	**-75.5**
CHD^{Max}-1	**-61.3**	MYC/CHD^{Max}-2	**-133.1**
CHD^{Max}-2	**-60.7**	MYC/CHD^{Max}-3	**-77.5**
CHD^{Max}-3	**-45.1**		

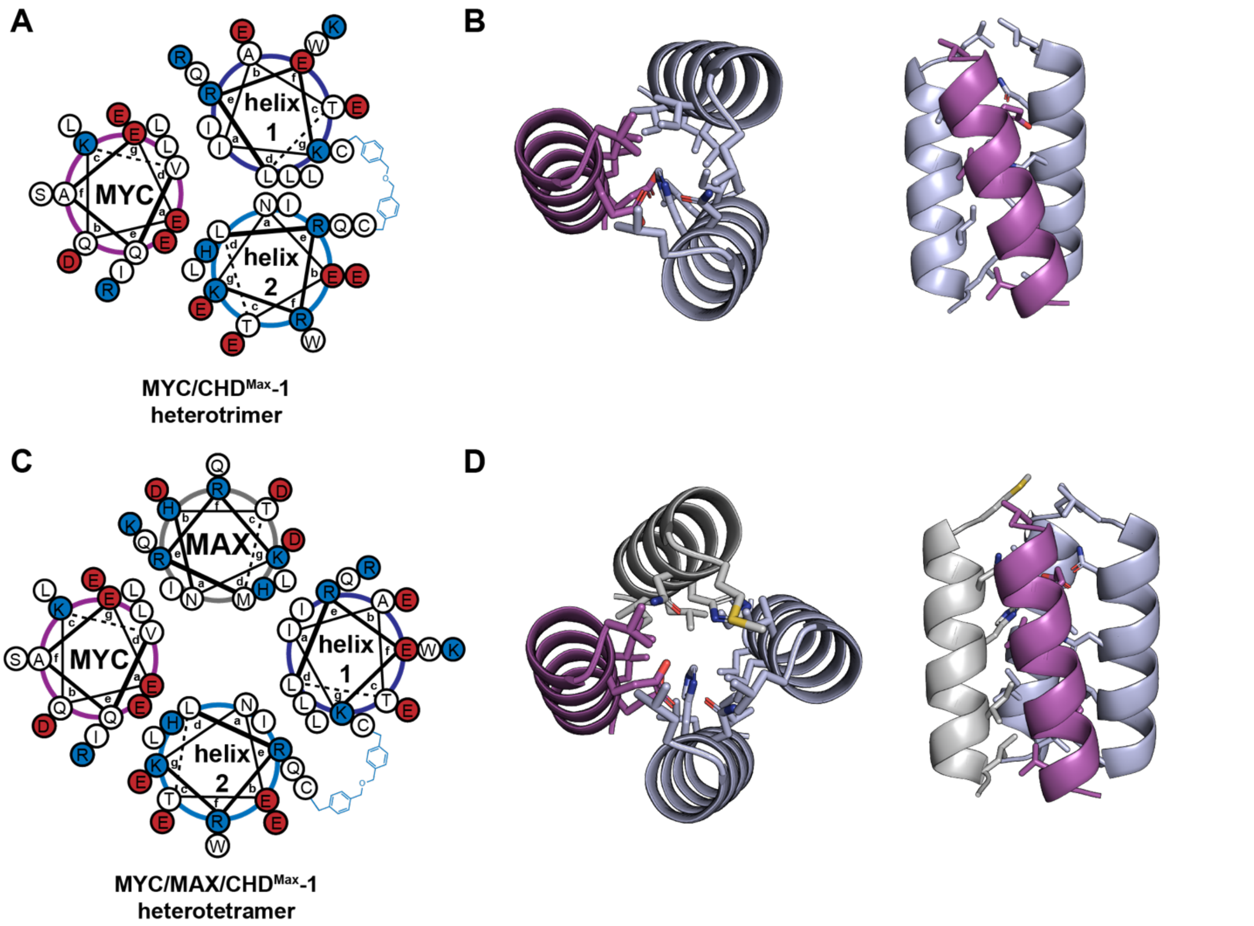

Figure S2. Modeling parallel heterotrimer and heterotetramer with **CHD^{Max}-1.** (A) Helical wheel representation of MYC/**CHD^{Max}-1** trimer. (B) Top-down view (left) and side on view (right) of MYC/**CHD^{Max}-1** trimer. (C) Helical wheel representation of MYC/MAX/**CHD^{Max}-1** tetramer. (D) Top-down view (left) and side on view (right) of MYC/MAX/**CHD^{Max}-1** tetramer. Positively charged residues are highlighted blue and negatively charged residues in red. Models produced in CCBuilder.

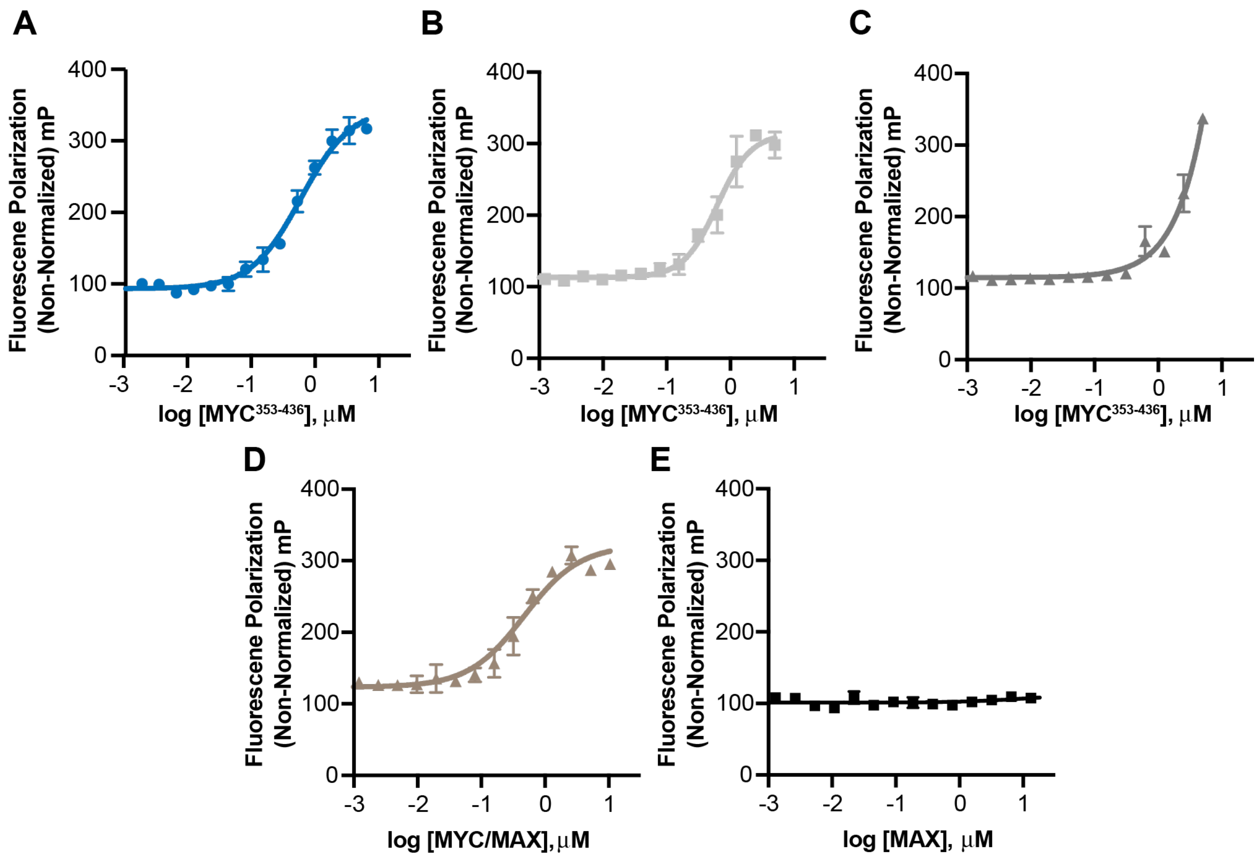

Figure S3. Non-normalized fluorescence polarization curves for binding of fluorescently labeled peptides. Fluorescence polarization assay to assess direct binding between fluorescein-labeled (A) **CHD^{Max}-1** and His$_6$-Myc$^{353\text{-}437}$ (B) **CHD^{Max}-2** and His$_6$-MYC$^{353\text{-}437}$ (C) **CHD^{Max}-3** and His$_6$-MYC$^{353\text{-}437}$ (D) **CHD^{Max}-1** and MYC/MAX complex (E) **CHD^{Max}-1** and His$_6$-MAX.

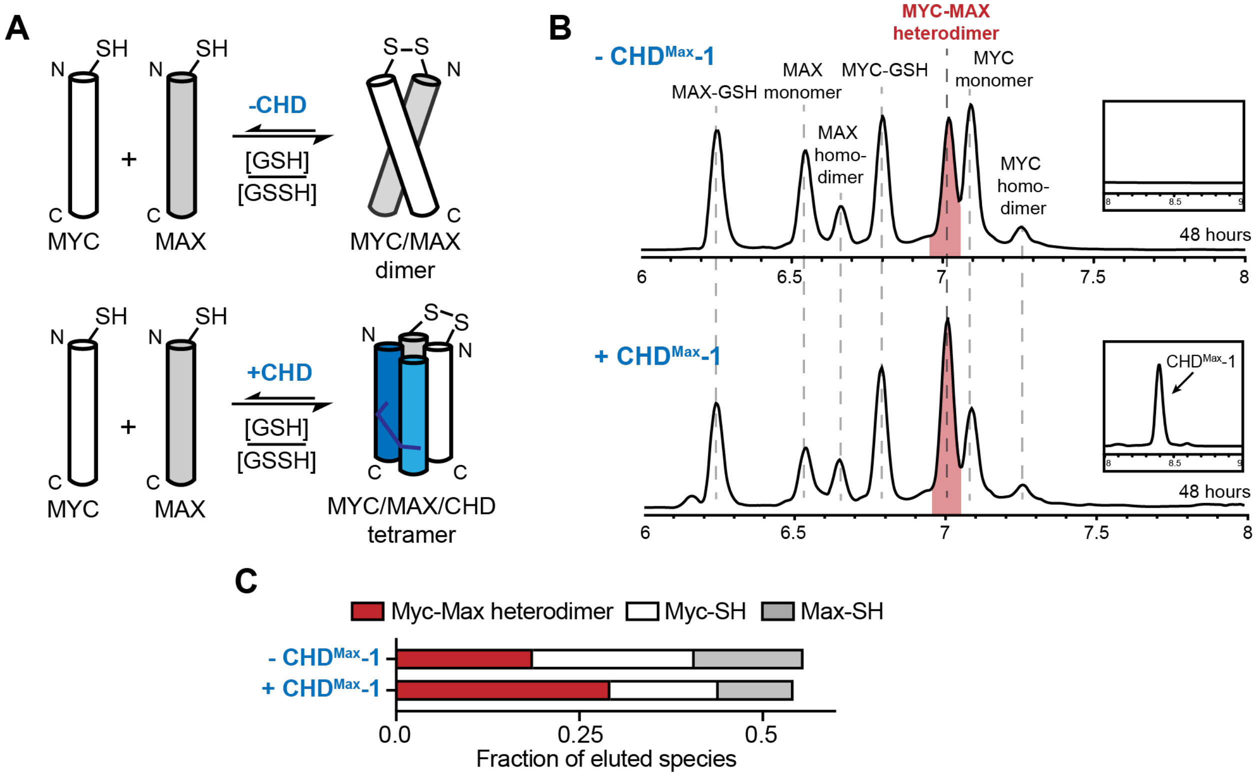

Figure S4. (A) Schematic representation of thiol-disulfide exchange assay in this study. (B) Overlay HPLC spectra after 48 hours of co-incubation of MYC-LZ/MAX-LZ with or without **CHD^{Max}-1** under redox conditions. (C) Fraction of eluted species quantified by HPLC indicates an increase in MYC/MAX dimer in the presence of **CHD^{Max}-1.**

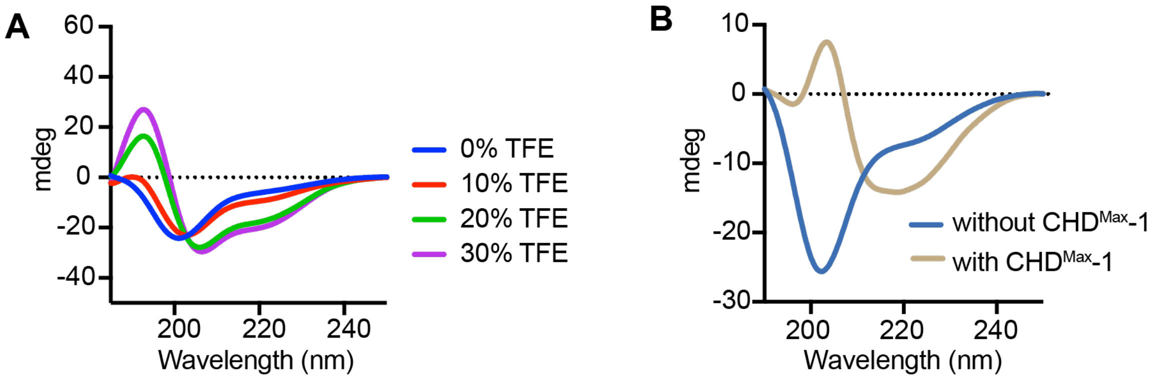

$MYC^{406-421}$	0% TFE	10% TFE	20% TFE	30% TFE
$\lvert mdeg_{222} \rvert$	5.74	8.89	17.29	19.61

$MYC^{406-421}$	without CHD^{Max}-1	with CHD^{Max}-1
$\lvert mdeg_{222} \rvert$	7.00	13.51

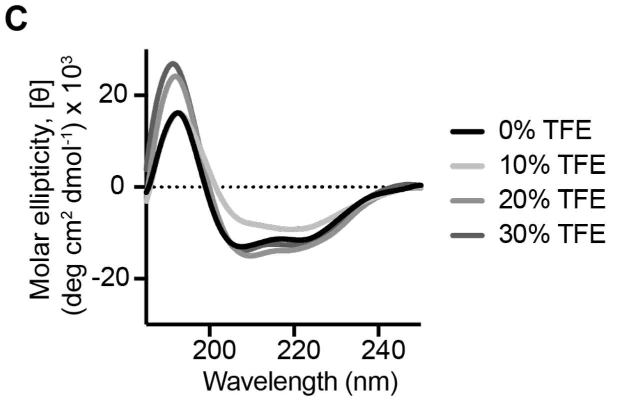

Figure S5. (A) (Top) CD spectra of 100 μM $MYC^{406-421}$ with different percentages of 2,2,2-trifluoroethanol (TFE) in 0.1X phosphate buffer saline (PBS) pH 6.7. (Bottom) mdeg values at 222 nm of $MYC^{406-421}$ at different percentage of TFE. (B) (Top) CD spectra of $MYC^{406-421}$ in the presence or absence of **CHD^{Max}-1**, calculated by subtracting **CHD^{Max}-1** values from the experiment sum in Figure 3B. (Bottom) Change in mdeg values at 222 nm indicates an increase in helicity of $MYC^{406-421}$ upon complexing with **CHD^{Max}-1.** (C) CD spectra of 20 μM **CHD^{Max}-1** with different percentages of TFE in 0.1X phosphate buffer saline (PBS) pH 6.7.

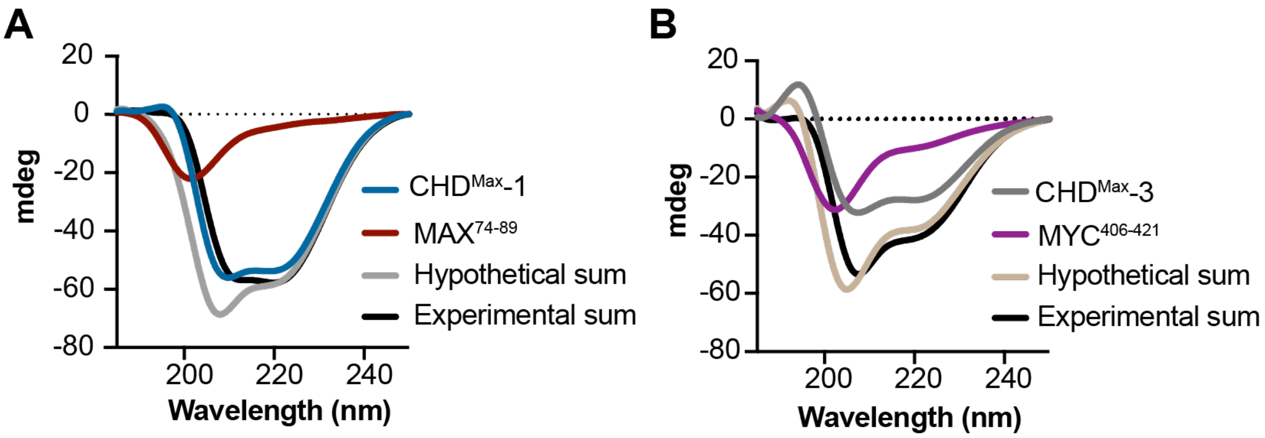

Figure S6. (A) CD spectra of **CHD^{Max}-1** co-incubated with MAX$^{74-89}$. Hypothetical sum (dark gray) indicates the sum of two independent spectra while experimental sum (black) represents mixture of co-titrated peptides. (B) CD spectra of **CHD^{Max}-3** co-incubated with MYC$^{406-421}$. Hypothetical sum (tan) indicates the sum of two independent spectra while experimental sum (black) represents mixture of co-titrated peptides. CD experiments were conducted in 0.1X phosphate buffer saline (PBS) pH 6.7 with 100 μM peptide concentration.

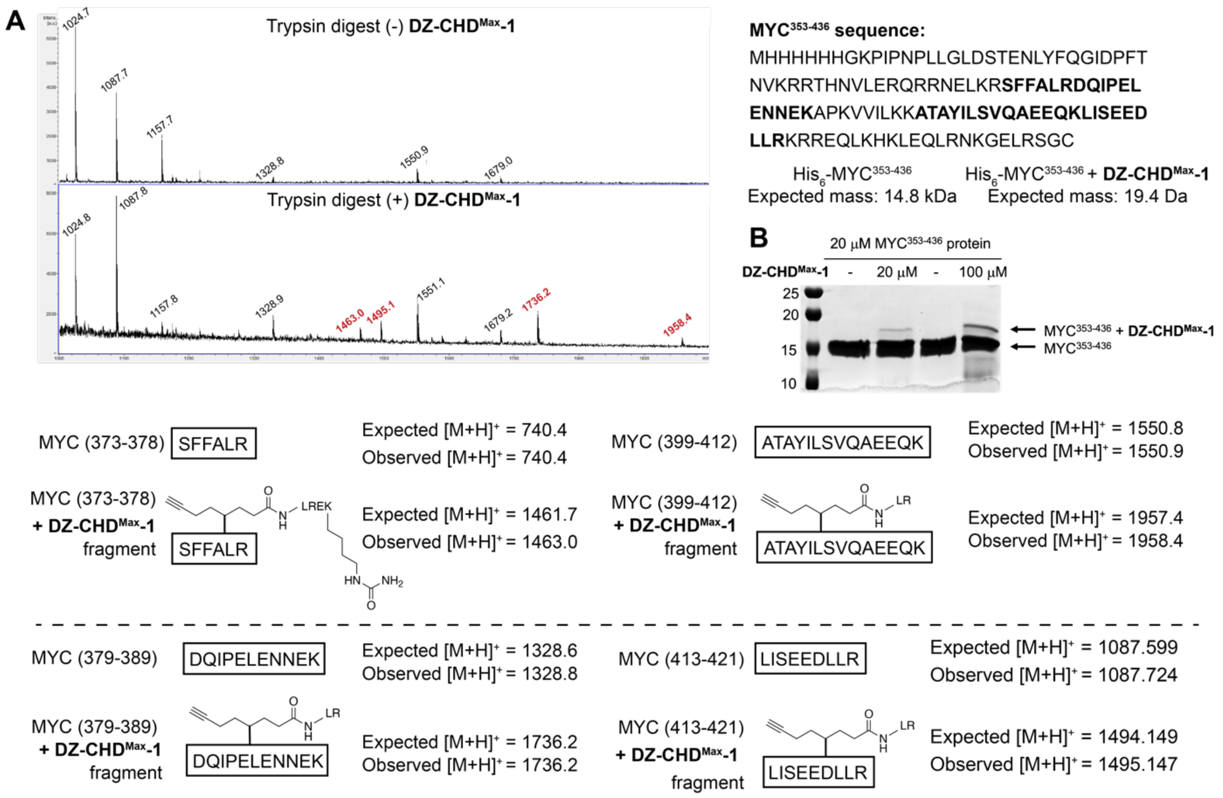

Figure S7. Chemical crosslinking of diazirine-labeled CHD^{Max}-1 reveals proximal binding sites on MYC. (A) MALDI-TOF spectra displaying identified fragment masses of trypsin-digested **DZ-CHD^{Max}-1** crosslinked to the MYC leucine zipper region. Corresponding labeled mass was not observed from the unlabeled MYC sample. (B) Gel shift assay of MYC incubated with and without **DZ-CHD^{Max}-1** at 20 μM and 100 μM concentration.

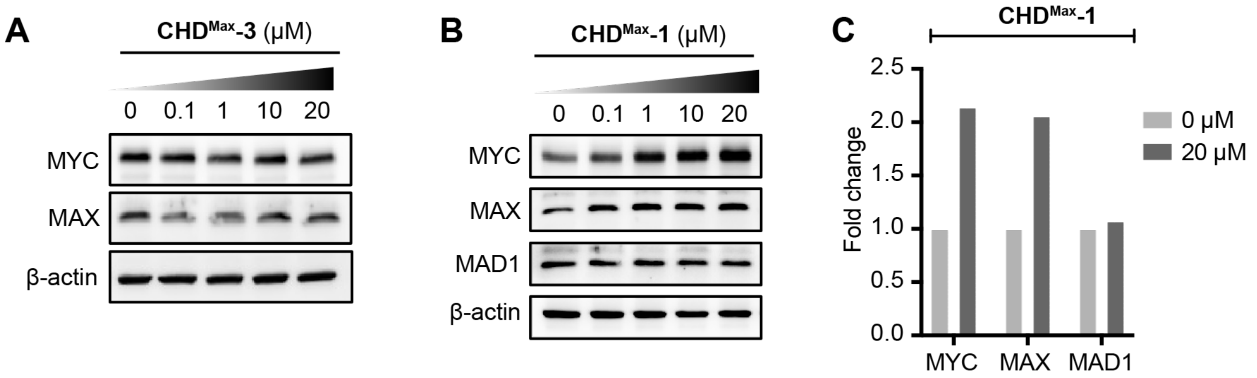

Figure S8. Representative Western blots of the effect of CHDs in cells. (A) MYC and MAX protein levels after treatment with **CHD^{Max}-3** for 24 hours in T24 cells. (B) MYC and MAX protein levels after treatment with **CHD^{Max}-1** for 24 hours in HCT-116 cells. (C) Relative protein levels of MYC/MAX/MAD1 normalized to β-actin from western blot in B.

Supporting Methods

General Information

T24 and HCT-116 cells were maintained in Dulbecco's modified eagle medium (DMEM, Gibco) supplemented with 10% fetal bovine serum (FBS, Gibco), 1X penicillin/streptomycin (Gibco), and 10 mM HEPES buffer. All cells were kept in a humidified incubator at 37°C and 5% CO_2.

Coiled coil modeling with CCBuilder 2.0

Initial structures were constructed from PDB of CC-Di/CC-Tri/CC-Tet obtained from CCBuilder2.0 using generalized parameters.[3] For parallel trimeric and tetrameric coiled coil complexes, we modeled and optimized for parameter space in ISAMBARD using metaheuristics and evo_optimizers module.[4] To assess the quality of a model, we relied on BUFF values (Bristol University Docking Engine Force Field), a stand-alone implementation of the all-atom force field from BUDE (Bristol University Docking Engine). The lowest energy models were then visualized using PyMol.

Table S3. Coiled coil parameters conducted in ISAMBARD and modeling scores.

Species	Radius	Pitch	Interface angle	BUDE energy	BUFF Score
MYC/CHD^{Max}-1	6.2	172.6	28	-161.9	-794.0
MYC/MAX/ CHD^{Max}-1	7.2	243.2	26	-230.4	-1029.3

Synthesis and Characterization of Crosslinked Helix Dimers[5]

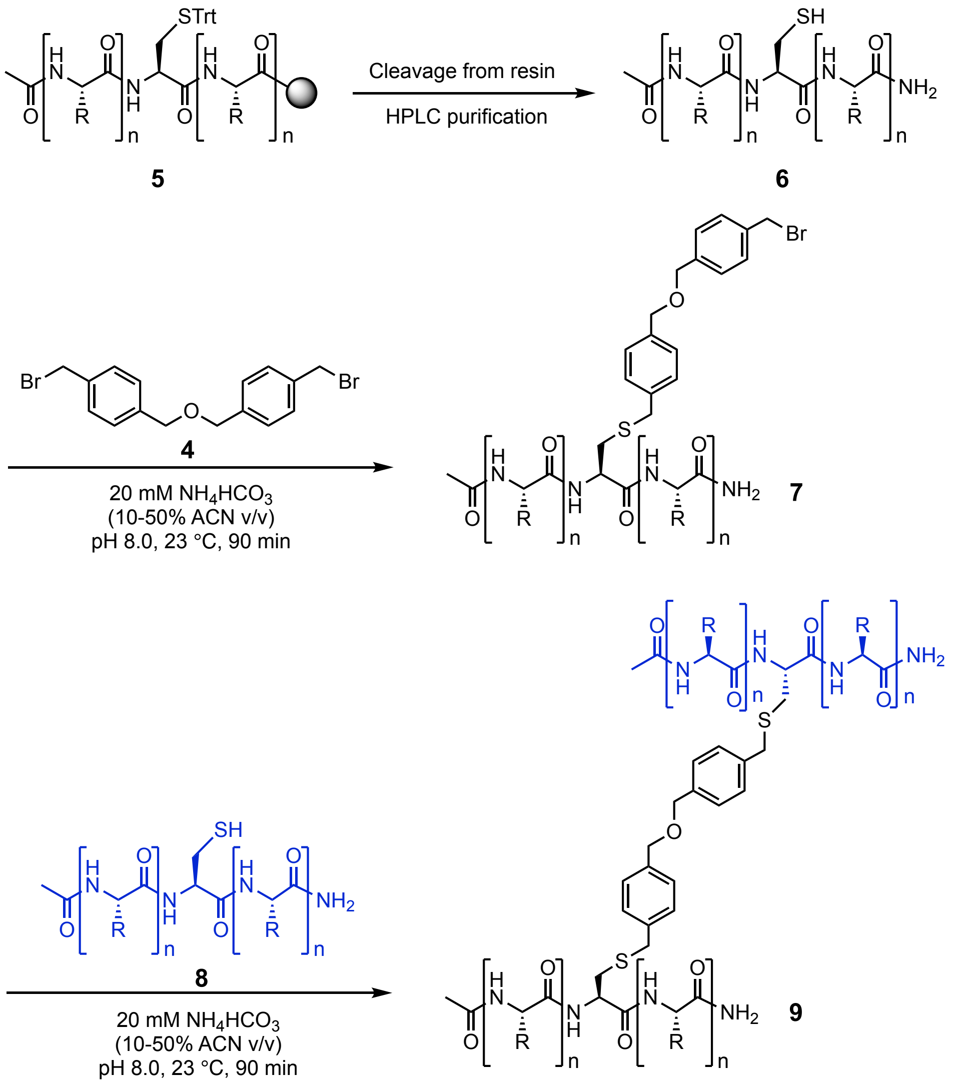

Scheme 1. Synthesis of dibenzyl ether CHDs

Synthesis of bis-triazole CHDs was performed using an established protocol.[5,6] Parent peptide strand **5** was synthesized using standard Fmoc solid-phase peptide synthesis on Rink Amide resin. After final Fmoc deprotection, the peptides were acetylated at the N terminus using a solution of 1 M acetic anhydride in DMF for 30 min prior to cleavage from resin. **5** was cleaved from resin using 94% trifluoroacetic acid, 2.5% EDT, 2.5% H2O and 1% TIPS, purified via reversed-phase HPLC to obtain **6** (gradient 25-70 acetonitrile/water with 0.1% TFA over 45 min), and characterized by MALDI-TOF spectrometry. Crosslinking reaction was performed as previously described. The first strand **6** was mono-alkylated with 5 equivalents of bis-bromo dibenzyl ether crosslinker **4**[5] in 20 mM NH_4HCO_3/acetonitrile pH 8.0. The reaction mixture was stirred for 90 min at 20 °C followed by purification via RP-HPLC to obtain **7** (gradient 25-70 acetonitrile/water with 0.1% TFA over 45 min). The purified product **7** was subsequently lyophilized prior to a second conjugation with an excess amount of the second strand **8** (1.5 eq.)

in 20 mM NH_4HCO_3/acetonitrile pH 8.0. The resulting mixture was stirred for 1 h at 20 °C followed by purification via RP-HPLC to obtain **9** (gradient 25-70 acetonitrile/water with 0.1% TFA over 45 min) and characterized by analytical HPLC and MALDI-TOF spectroscopy.

Synthesis of Fluorescein-labeled CHDs

The listed peptide sequences were synthesized as previously described with β-alanine added to the N terminus to serve as a linker prior to coupling with fluorescein isothiocyanate (3 eq.) and DIEA (3 eq.) overnight and protected from light. The fluorophore-conjugated peptides were then cleaved from resin, purified via RP-HPLC, and characterized with MALDI-TOF spectrometry.

Synthesis of Diazirine-conjugated CHD

The listed peptide sequences were subjected to coupling with 3-(3-(but-3-ynyl)-3H-diazirin-3-yl)propanoic acid (Sigma-Aldrich) (3 eq.) and DIEA (3 eq.) overnight and protected from light. The fluorophore-conjugated peptides were then cleaved from resin, purified via RP-HPLC, and characterized with MALDI-TOF spectrometry.

Synthesis of Biotin-conjugated CHD

The listed peptide sequences were subjected to coupling with D-biotin (Chem-Impex) (5 eq.), HOBt (5 eq.), DIC (5 eq.) in NMP. The biotin-conjugated peptides were then cleaved from resin, purified via RP-HPLC, and characterized with MALDI-TOF spectrometry.

CHD^{Max}-1

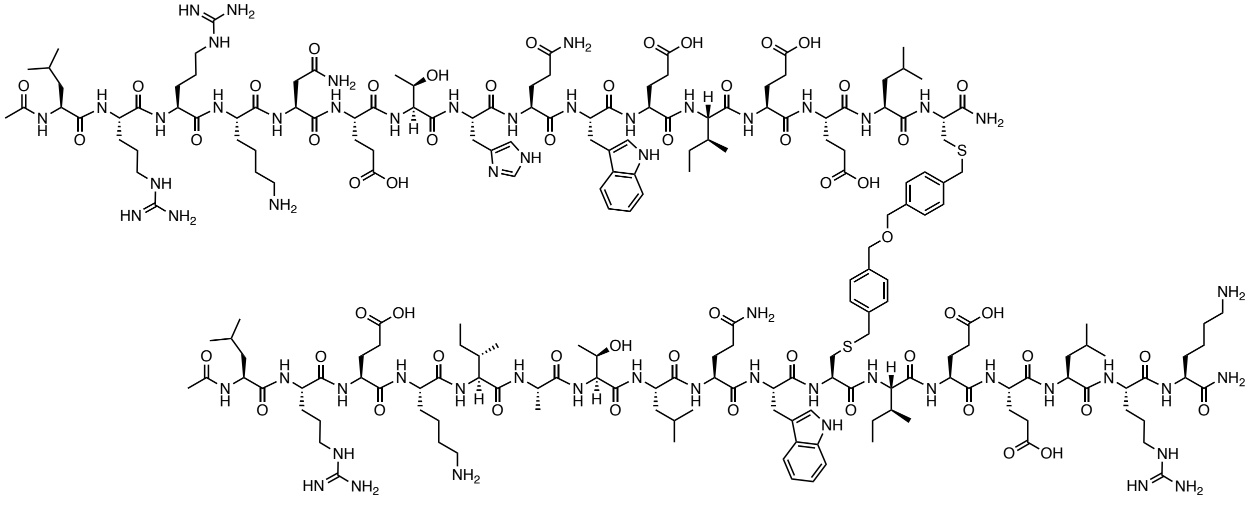

FITC-CHD^{Max}-1

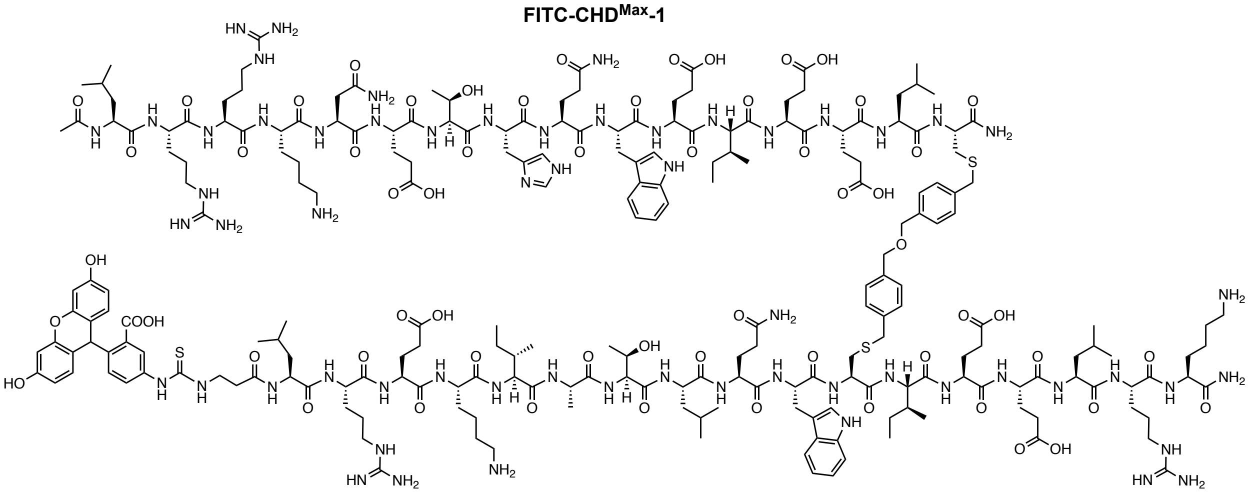

DZ-CHD^{Max}-1

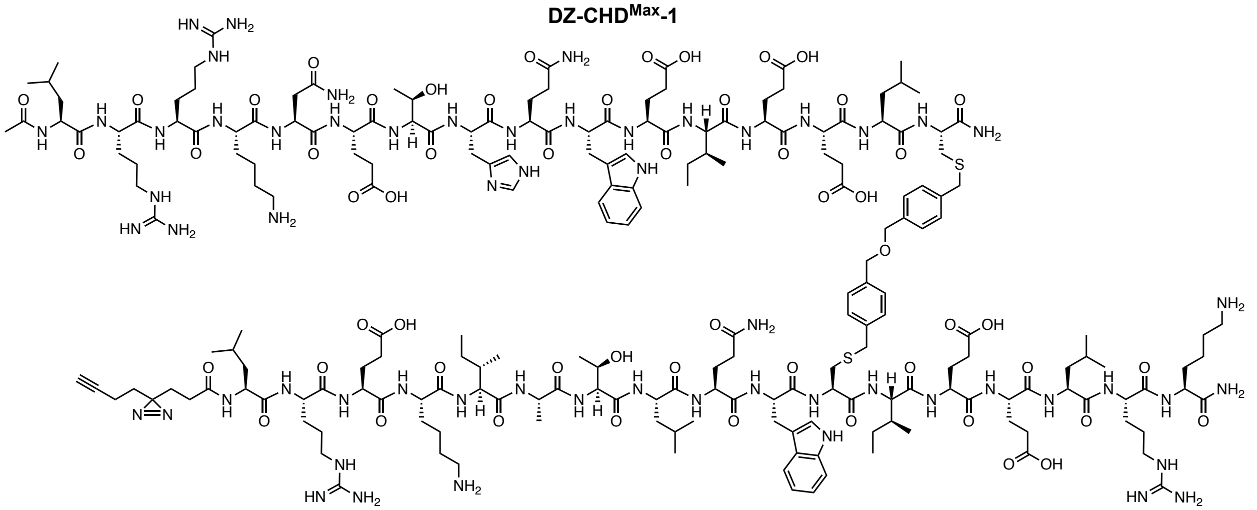

Biotin-PEG$_2$-CHD^{Max}-1

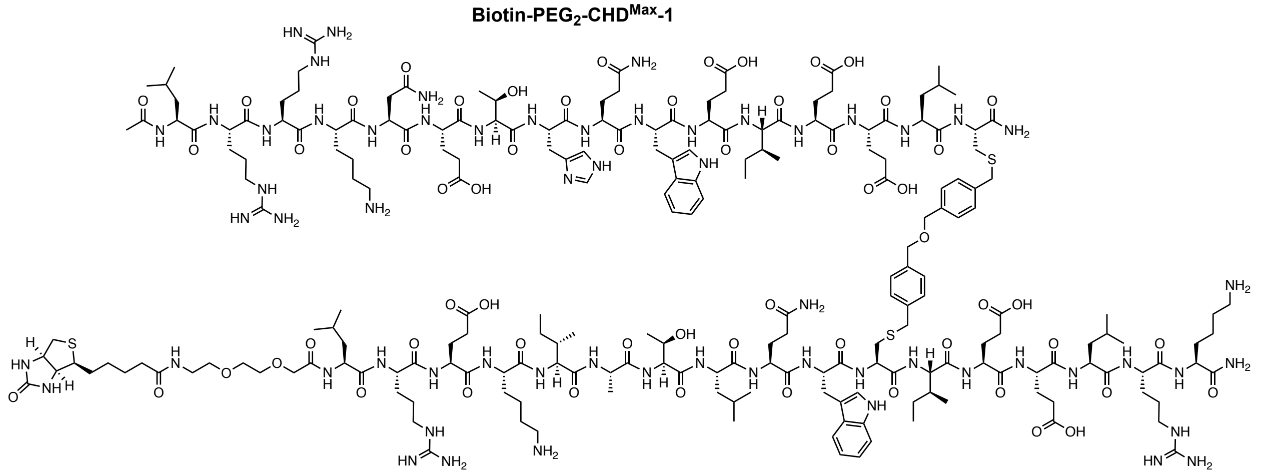

CHD^{Max}-2

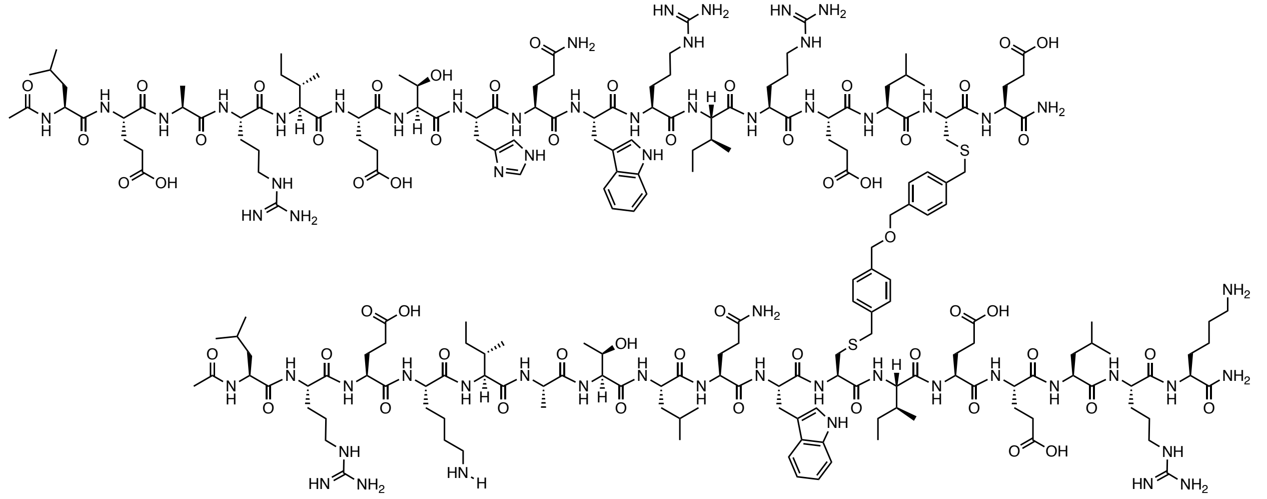

FITC-CHD^{Max}-2

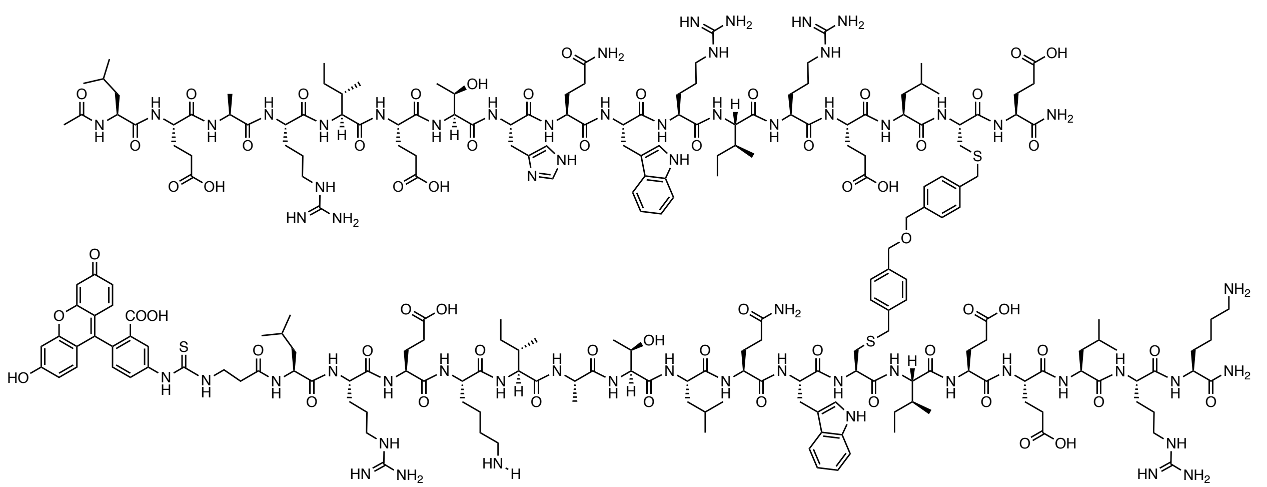

CHD^{Max}-3

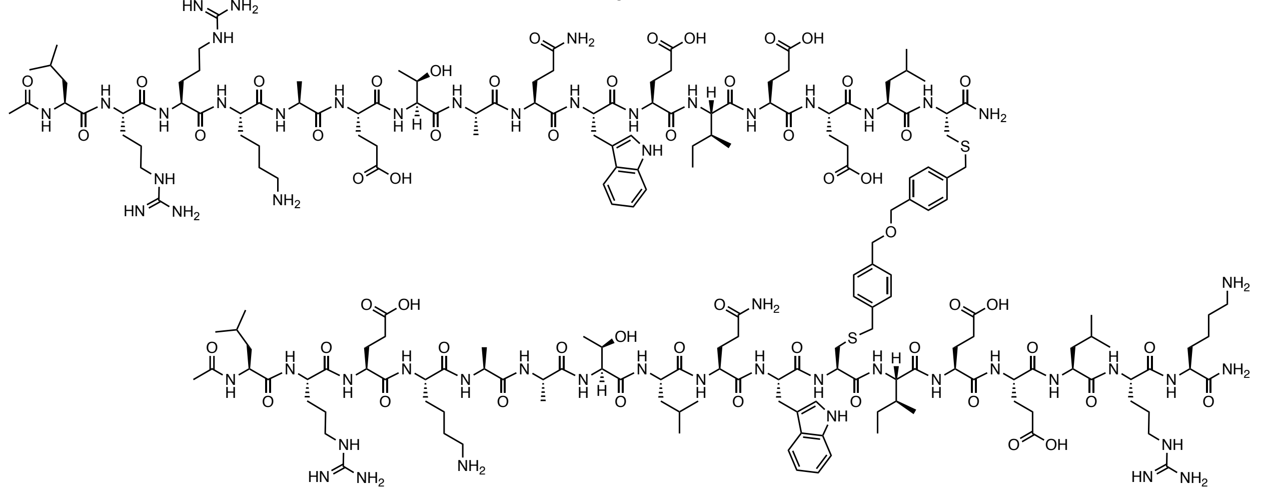

FITC-CHD^{Max}-3

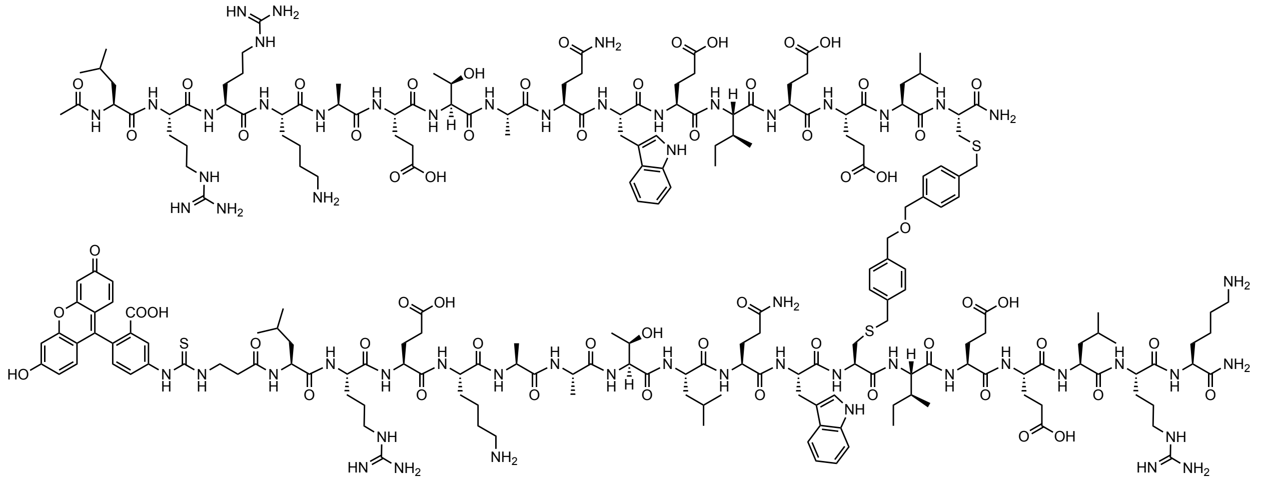

Figure S9. Chemical structures of Max CHDs.

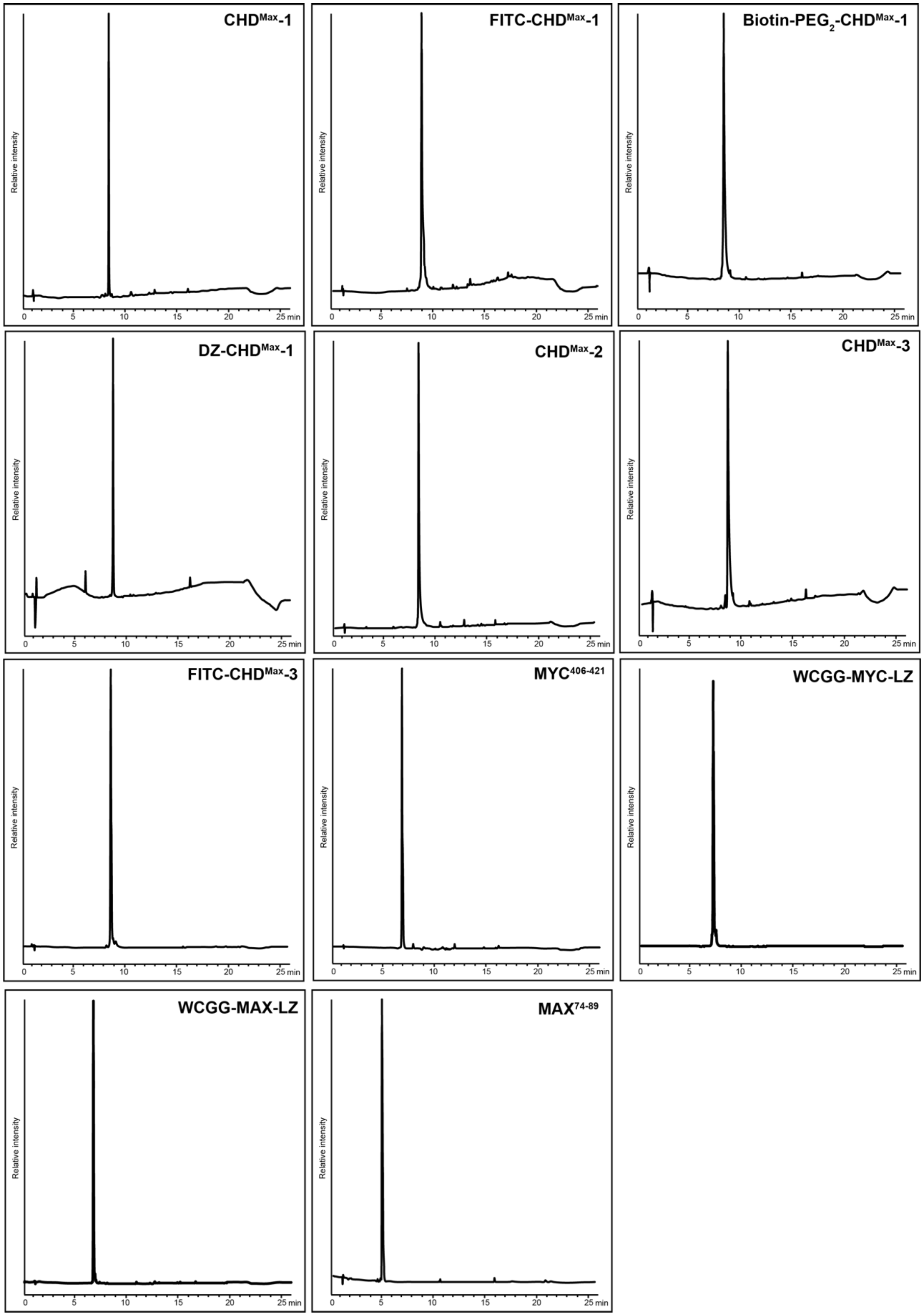

Figure S10. Analytical HPLC traces of purified peptides.

Table S4. Mass spectroscopic characterization of CHD peptides.
A$_{\beta}$ = L-β-alanine; DZ = diazirine photocrosslinker; FITC is 5-fluorescein isothiocyanate linked via thiourea bond to N-terminal amine. DABCYL is 4-((4-(dimethylamino)phenyl)azo)benzoic acid amide linked to N-terminal amine. THAL is 2-(2,6-Dioxopiperidin-3-yl)-1,3-dioxoisoindoline-5-carboxylic acid linked via amide bond to N-terminal amine.

Compound	Helix name	Sequence	Calculated (M+H)$^+$	Observed (M+H)$^+$
CHD^{Max}-1	1d	Ac-LREKIATLQWCIEELRK-NH$_2$	4518.3	4518.6
	2d	Ac-LRRKNETHQWEIEELC-NH$_2$		
CHD^{Max}-2	1d	Ac-LREKIATLQWCIEELRK-NH$_2$	4616.4	4615.4
	2e	Ac-LEARIETHQWRIRELCE-NH$_2$		
CHD^{Max}-3	1e	Ac-LREKAATLQWCIEELRK-NH$_2$	4367.1	4368.5
	2f	Ac-LRRKAETAQWEIEELC-NH$_2$		
FITC-CHD^{Max}-1	FITC-1d	FITC-A$_{\beta}$LREKIATLQWCIEELRK-NH$_2$	4936.7	4936.7
	2d	Ac-LRRKNETHQWEIEELC-NH$_2$		
FITC-CHD^{Max}-2	FITC-1d	FITC-A$_{\beta}$LREKIATLQWCIEELRK-NH$_2$	5034.9	5033.4
	2e	Ac-LEARIETHQWRIRELCE-NH$_2$		
FITC-CHD^{Max}-3	FITC-1e	FITC-A$_{\beta}$LREKAATLQWCIEELRK-NH$_2$	4785.5	4785.5
	2f	Ac-LRRKAETAQWEIEELC-NH$_2$		
DZ-CHD^{Max}-1	DZ-1d	DZ-LREKIATLQWCIEELRK-NH$_2$	4596.4 (- N$_2$)	4600.0
	2d	Ac-LRRKNETHQWEIEELC-NH$_2$		
Biotin-PEG$_2$-CHD^{Max}-1	biotin-PEG$_2$-1d	Biotin-PEG$_2$-LREKIATLQWCIEELRK-NH$_2$	4847.7	4849.2
	2d	Ac-LRRKNETHQWEIEELC-NH$_2$		
MYC-LZ	WCGG-MYC-LZ	Ac-WCGGVQAEEQKLISEEDLLRKRREQLKHKLEQL-NH$_2$	4032.6	4033.2
MAX-LZ	WCGG-MAX-LZ	Ac-WCGG(Nle)RRKNDTHQQDIDDLKRQNALLEQQVRAL-NH$_2$	3960.4	3960.9
MYC$^{406-421}$		Ac-YA$_{\beta}$VQAEEQKLISEEDLLR-NH$_2$	2175.4	2175.6
MAX$^{74-89}$		Ac-Y(Nle)RRKNHTHQQDIDDLK-NH$_2$	2221.4	2220.6

Protein Purification

pET151-D-TOPO-cMyc vector was kindly provided by Dr. Edward Prochownik from the Department of Pediatrics at UPMC Children's Hospital of Pittsburgh. Expression and purification of $Myc^{353-437}$ were performed using an established protocol with minor modifications.[7] BL21(DE3) cells were used to express recombinant $Myc^{353-437}$ protein. Bacteria were grown at 37 °C to OD600 of 0.8 followed by 1 mM IPTG induction and 16-18h growth at RT. Cells were harvested via centrifugation at 4,000 rpm at 4 °C and the pellet was resuspended in 10 mL of lysis buffer (8 M urea, 100 mM NaH_2PO_4, 10 mM Tris HCl, pH 8) supplemented with protease inhibitor cocktail (Pierce™ Protease Inhibitor Tablets) and benzonase nuclease. Suspensions were sonicated on ice using a Branson Cell Disruptor 200 and centrifuged at 12,000 rpm for 45 min at 4 °C. Clarified lysate was applied to a column containing pre-equilibrated HisPur Ni-NTA Superflow Agarose (ThermoFisher Scientific) for overnight at 4 °C. The resin beads were washed with 10 mL of additional lysis buffer followed by three washes of 10 mL wash buffer (8 M urea, 100 mM NaH_2PO_4, 10 mM Tris HCl, 10 mM imidazole). The target protein was eluted with elution buffer (8 M urea, 100 mM NaH_2PO_4, 10 mM Tris HCl, 250 mM imidazole) and buffer exchanged into 150 mM NaCl, 50 mM Tris HCl, 5% glycerol, pH 6.7. Protein was concentrated by ultra-filtration (3000 MWCO; Amicon Ultra-15 Centrifugal Filter units from EMD Millipore) followed by TEV cleavage (1:100 w/w of protease to target protein) in 150 mM NaCl, 50 mM Tris HCl, 5% glycerol, pH 6.7 for 24 hrs at 4 °C. The cleaved protein is further purified by reverse-phase HPLC (gradient 10-60 acetonitrile/water with 0.1% TFA over 35 min) to remove TEV protease and un-cleaved protein. Purified $Myc^{353-437}$ was lyophilized and stored at -20 °C. $Myc^{353-437}$ identity was characterized by SDS-PAGE and MALDI-TOF.

Fluorescence Polarization Binding Assay

The relative affinities of FITC-CHD^{Max}-1 to His_6-$Myc^{353-436}$ and full-length His_6-Max were determined using fluorescence polarization-based direct binding assay. The polarization experiments were performed using a DTX 880 Multimode Detector (Beckman) at 25 °C with excitation and emission wavelengths of 485 and 525 nm, respectively. Each polarization experiment was performed in a 96-well round bottom plate (Greiner) in assay buffer: 1X PBS, 1 mM DTT, 5% glycerol, 0.1% pluronic acid, pH 6.7. The binding affinity (K_D) values reported for each peptide are from experiments performed in triplicate. Raw values were fit to a sigmoidal dose-response nonlinear regression model in GraphPad Prism 6.0.

All binding experiments with FITC-labeled peptides to His_6-$Myc^{353-436}$ or His_6-Max were performed under the same conditions. Briefly, serial dilutions of His_6-$Myc^{353-436}$ and His_6-Max were made from 10 μM into 15 nM of FITC-labeled peptide in assay buffer. K_D was determined using the following equation:

$$K_{D1} = (R_T*(1-F_{SB}) + L_{ST}*F_{SB}^2)/F_{SB}-L_{ST}$$

where,

R_T = Total concentration of His_6-$Myc^{353-436}$ or His_6-Max

L_{ST} = Total concentration of FITC-labeled peptide

F_{SB} = Fraction of bound FITC-labeled peptide

Circular Dichroism

CD experiments were conducted on an Jasco J-1500 Circular Dichroism spectrometer equipped with a temperature controller using 1 mm length cells and a scan speed of 4.0 nm/min at 298 K. The spectra were averaged over 5 scans with baseline subtraction. Raw values were normalized to molar residue ellipticity. Each sample was prepared in 0.1X phosphate buffer saline (PBS) pH 6.7 to a final concentration of 20 µM. The concentrations of each peptide were determined by the UV absorption at 280 nm.

Serum Stability Assay

Proteolytic stability of CHD^{Max}-1 was assessed in 25% serum by using fetal bovine serum (FBS, Gibco) in Roswell Park Memorial Institute (RPMI)-1640 medium at 37 °C. A 90 µL mixture of 33% FBS (v/v) in serum-free medium was prepared for each sample. The addition of 30 µL indicated peptide in serum-free medium (25% FBS final) per each experiment was recorded as the starting time point. Time points of 0 h, 6 h, and 24 h were analyzed in triplicate. Each experiment was quenched at the determined time point by adding 60 µL 100% EtOH, which was pre-cooled at −70 °C. After cooling on ice for 10 min, each sample was pelleted at 14,000 × g for 5 min. A total of 180 µL supernatant was isolated, and 18 µL 500 µM L-Tryptophan was titrated into the sample to be used as an internal standard. The resulting mixture was then immediately subjected to RP-HPLC with a C18 3.5 µm 2.1 × 150 mm analytical column and monitored at 280 nm. Eluting peaks were collected, and the mass of each peak was determined using MALDI-TOF spectroscopy. Integrated peak area of nondegraded peptides was used to determine the percent surviving in each given condition.

Thiol-disulfide equilibrium exchange

MYC-LZ and MAX-LZ peptides containing N-terminal cysteines were reconstituted to a final concentration of 100 µM in 50 mM Tris, 150 mM NaCl, pH 7.4 supplemented with 500 µM of reduced glutathione (GSH) and 250 µM of oxidized glutathione (GSSG). Reaction mixture was allowed to equilibrate overnight at on an orbital shaker at 25 °C. At each indicated timepoint, reaction mixture was separated by analytical RP-HPLC using an InfinityLab Poroshell 120 EC-C18, 2.7 µm, 4.6 x 100 mm column (Part No. 695975-902).

***In Vitro* Pulldown Assay**

3.0 x 10^6 T24 cells were washed with ice-cold PBS and lysed in cold buffer A (10 mM HEPES, 10 mM KCl, 1.5 mM $MgCl_2$, 0.2 mM EDTA, 1 mM DTT, 0.5% NP40, pH 7.9, cOmplete™ Protease Inhibitor Cocktail (Roche) and Halt™ Phosphatase Inhibitor Cocktail (Thermo)). Lysates were incubated on ice for 20 min and nuclei were pelleted by centrifugation. Supernatants containing cytoplasmic proteins were collected and stored at -80 °C. Pellets were washed once with buffer B (10 mM HEPES, 10 mM KCl, 1.5 mM $MgCl_2$, 0.2 mM EDTA, 1 mM DTT, pH 7.9, cOmplete™ Protease Inhibitor Cocktail (Roche) and Halt™ Phosphatase Inhibitor Cocktail (Thermo)) and resuspended in buffer C (20 mM HEPES, pH 7.9, with 1.5 mM $MgCl_2$, 0.42 M NaCl, 0.2 mM EDTA, 25% (v/v) Glycerol, 1 mM DTT, cOmplete™ Protease Inhibitor Cocktail (Roche) and Halt™ Phosphatase Inhibitor Cocktail (Thermo)), incubated on ice for 60 min with vortexing every 10 min. Supernatants containing nuclear proteins were collected after centrifugation and stored at -80 °C. Protein lysates were dialyzed into 20 mM HEPES, 150 mM NaCl and 1.5 mM $MgCl_2$ pH 8.0 for 2 hours at 4 °C and pre-cleared with Dynabead™ M-280 Streptavidin (Thermo)

for 1 hr at 4 °C. Protein lysates were quantified with Pierce BCA Assay (Thermo). 200 μg of the cleared nuclear extract was incubated with 20, 10, or 1 μM biotinylated CHDs on a rotator overnight at 4 °C. Next day, 60 μL of streptavidin beads was added to each sample and further rotated for 60 min at 4 °C. Beads were washed with buffer C containing 0.1% BSA for 3 times and another 3 times with buffer C without BSA, then eluted with 2X sample buffer and boiled at 95 °C for 5 min. The supernatant was subjected to Western Blot for pulldown analysis.

Quantification of cellular level of MYC, MAX, MAD1

The indicated cells were initially seeded in 6-well plates (0.3 x 10^6 cells/well) and allowed to attach overnight. The cells were then treated with indicated peptides or DMSO dissolved in serum-free medium (1% DMSO *v/v*) at 0.1, 1, 10, or 20 μM for 4 hours at 37 °C followed by addition of 10% FBS and 16 hours of incubation. After the indicated time, cells were washed with ice-cold PBS then extracted using RIPA buffer (Pierce) supplemented with cOmplete™ Protease Inhibitor Cocktail (Roche) and Halt™ Phosphatase Inhibitor Cocktail (Thermo) followed by incubation for 20 minutes on ice. The resulting lysate was centrifuged at 13,000 rpm for 10 minutes at 4 °C. The clarified lysates were collected and quantified with Pierce BCA Assay (Thermo). Relative protein levels were determined via immunoblotting, using anti-c-Myc, anti-Max, anti-Mad and β-actin antibodies described in Table S5.

Table S5. Resources table

**Reagent or resource**	**Source**	**Identifier**
**Antibodies**		
Rabbit mAb to c-Myc (Y69)	Abcam	ab32072
Rabbit pAb to Max (S20)	Cell Signaling Technology	4739S
Rabbit pAb to Mad-1	Cell Signaling Technology	4682S
Rb mAb to β-actin (13E5)	Cell Signaling Technology	4970T
Goat Anti-Rabbit IgG-HRP Conjugate	Cell Signaling Technology	7074P2

Live-Cell Fluorescence Microscopy

The indicated cell lines were seeded at 1 × 10^5 cells/well in poly-D-lysine–coated 35 mm plates (MatTek) and incubated overnight. The growth medium is aspirated and washed with serum-free medium. The cells are then incubated with 1 μM (final) of fluorescein-conjugated peptides dissolved in serum-free medium (0.4% DMSO v/v) for 4 hours at 37°C while protected from light. All compounds are dissolved as concentrated stocks in DMSO. After the specified incubation time, each plate was aspirated and treated with Hoechst dye solution (Hoechst 33342, ThermoFisher Scientific) for 10 minutes to stain cell nuclei. The Hoechst dye stock solution (10 mg/mL) was diluted 1:2000 in PBS to form the working mixture. The dye solution was removed, and the plate was gently washed 3x with PBS. DMEM (high glucose, HEPES, no phenol red, 20%

FBS) was added to each plate and used as the imaging solution. Fluorescence images were acquired on a Leica SP8 confocal microscope equipped with 63X objective lens.

Flow cytometry

The indicated cell lines were seeded at 1 x 10^5 cells/well in clear polystyrene 24-well plates (Corning) and incubated overnight. The initial growth media is replaced with serum-free media and incubated for 2 hours at 37 °C. Upon aspiration, the cells are incubated in serum-free media (0.4% DMSO v/v) for one hour. The cells were then treated with 1 μM (final) of fluorescein-conjugated peptides in serum-free media for another hour while protected from light. All compounds are dissolved as concentrated stocks in DMSO. Each well was aspirated and treated with 1X trypsin (0.25% trypsin, 2.21 mM EDTA, Corning Cellgro) for 10 minutes at 37 °C. After trypsinization, the resulting solution was mixed with cold serum-free media and collected. The samples are centrifuged at 500 rpm for 5 minutes at 4 °C. The supernatant was removed, and the cell pellets were resuspended with cold PBS before placed on ice. Each sample was treated with 10% trypan blue (v/v) immediately before analysis by flow cytometry on a Cytek Aurora flow cytometer. The presented data consists of the mean fluorescence intensities for at least 10,000 cells/sample and processed using SpectroFlo (Cytek).

References

(1) Kortemme, T.; Kim, D. E.; Baker, D. Computational alanine scanning of protein-protein interfaces. *Sci STKE* **2004**, *2004*, pl2.

(2) Wood, C. W.; Ibarra, A. A.; Bartlett, G. J.; Wilson, A. J.; Woolfson, D. N.; Sessions, R. B. BAlaS: fast, interactive and accessible computational alanine-scanning using BudeAlaScan. *Bioinformatics* **2020**, *36*, 2917-2919.

(3) Wood, C. W.; Woolfson, D. N. CCBuilder 2.0: Powerful and accessible coiled-coil modeling. *Protein Sci* **2018**, *27*, 103-111.

(4) Wood, C. W.; Heal, J. W.; Thomson, A. R.; Bartlett, G. J.; Ibarra, A. A.; Brady, R. L.; Sessions, R. B.; Woolfson, D. N. ISAMBARD: an open-source computational environment for biomolecular analysis, modelling and design. *Bioinformatics* **2017**, *33*, 3043-3050.

(5) Hong, S. H.; Nguyen, T.; Arora, P. Design and Synthesis of Crosslinked Helix Dimers as Protein Tertiary Structure Mimics. *Curr Protoc* **2022**, *2*, e315.

(6) Wuo, M. G.; Hong, S. H.; Singh, A.; Arora, P. S. Synthetic Control of Tertiary Helical Structures in Short Peptides. *J Am Chem Soc* **2018**, *140*, 16284-16290.

(7) Jung, K. Y.; Wang, H.; Teriete, P.; Yap, J. L.; Chen, L.; Lanning, M. E.; Hu, A.; Lambert, L. J.; Holien, T.; Sundan, A.; Cosford, N. D.; Prochownik, E. V.; Fletcher, S. Perturbation of the c-Myc-Max protein-protein interaction via synthetic alpha-helix mimetics. *J Med Chem* **2015**, *58*, 3002-24.
